# Structural basis of protein condensation on microtubules underlying branching microtubule nucleation

**DOI:** 10.1038/s41467-023-39176-z

**Published:** 2023-06-21

**Authors:** Changmiao Guo, Raymundo Alfaro-Aco, Chunting Zhang, Ryan W. Russell, Sabine Petry, Tatyana Polenova

**Affiliations:** 1 Department of Chemistry and Biochemistry, University of Delaware, Newark, DE 19716 USA; 2 Department of Molecular Biology, Princeton University, Princeton, NJ 08544 USA

**Keywords:** Cytoskeletal proteins, Solid-state NMR

## Abstract

Targeting protein for Xklp2 (TPX2) is a key factor that stimulates branching microtubule nucleation during cell division. Upon binding to microtubules (MTs), TPX2 forms condensates via liquid-liquid phase separation, which facilitates recruitment of microtubule nucleation factors and tubulin. We report the structure of the TPX2 C-terminal minimal active domain (TPX2^α5-α7^) on the microtubule lattice determined by magic-angle-spinning NMR. We demonstrate that TPX2^α5-α7^ forms a co-condensate with soluble tubulin on microtubules and binds to MTs between two adjacent protofilaments and at the intersection of four tubulin heterodimers. These interactions stabilize the microtubules and promote the recruitment of tubulin. Our results reveal that TPX2^α5-α7^ is disordered in solution and adopts a folded structure on MTs, indicating that TPX2^α5-α7^ undergoes structural changes from unfolded to folded states upon binding to microtubules. The aromatic residues form dense interactions in the core, which stabilize folding of TPX2^α5-α7^ on microtubules. This work informs on how the phase-separated TPX2^α5-α7^ behaves on microtubules and represents an atomic-level structural characterization of a protein that is involved in a condensate on cytoskeletal filaments.

## Abstract (web summary)

TPX2 is a key factor stimulating branching microtubule (MT) nucleation. TPX2 forms condensates on MTs critical for branching. In this work, the authors report the atomic-level structure of TPX2 C-terminal minimal active domain on MT lattice and its binding interface, determined by magic-angle-spinning NMR.

## Introduction

The microtubule (MT) cytoskeleton regulates fundamental cellular processes by maintaining cell shape, organizing cellular interior, segregating chromosomes and providing long tracks for cargo transport. These functions require higher-order assembly of MTs that are tailored into specific cellular contexts, which relies on a plethora of microtubule-associated proteins (MAPs). MAPs are known to control MT nucleation, stability, dynamics and transport, as well as interactions between the MT cytoskeleton and other cellular components. In order to perform those functions, MAPs specifically recognize the MT lattice; however, atomic-level structural information on how MAPs bind to microtubules remains limited. Here, we structurally characterize a distinct type of interaction between a protein with intrinsically disordered regions (IDRs) and microtubules via condensation. Liquid–liquid phase separation (LLPS) is a mechanism that underlies the formation of membraneless organelles that compartmentalize cells^1^. Whereas many LLPS systems have been characterized at a cellular level and described in solution, it remains to be uncovered how they form and function on cellular surfaces such as MTs. Multiple MAPs have been shown to form single-component or multicomponent condensates on MTs^2–5^, but further understanding of these processes requires unveiling detailed structural and dynamic properties of these distinct protein assemblies.

During cell division, the MT cytoskeleton forms mitotic or meiotic spindles that drive chromosome segregation. A key player in spindle assembly is the multifunctional protein TPX2^6,7^, which stimulates the formation of the majority of spindle MTs via branching MT nucleation^8^. In this pathway, MTs nucleate from the side of pre-existing MTs, thus amplifying MT number while preserving their polarity^9^. TPX2 forms the linchpin of this reaction, as it needs to bind to MTs first in order to assemble the branching site. Disruption of TPX2’s function or expression levels causes spindle defects and genomic instability^6,10,11^, leading to tumorigenesis and metastasis of various human cancers, and may represent a prognostic biomarker for malignancies^12^.

An essential TPX2 region is its C-terminal part comprising domains α5, α6, and α7 (amino acids 477–716), which represents the minimal region of TPX2 capable of stimulating branching microtubule nucleation^13,14^. This minimal TPX2 part contains predicted alpha-helical regions and multiple regions homologous to nucleation activator motifs for the universal nucleator gamma-tubulin ring complex (γ-TuRC)^13^. These include a γ-TuRC nucleation activator motif (γ-TuNA) and the so-called SPM motif, which in TPX2 overlap with one another. The structure of these motifs and how they could potentially stimulate γ-TuRC is poorly understood. TPX2’s C-terminus interacts with the tetrameric kinesin Eg5/Kif11, which slides antiparallel MTs apart during spindle assembly^15^, while TPX2’s N-terminus interacts with the key mitotic kinase Aurora A^6,16–19^.

Essential for TPX2’s function is its ability to bind to MTs, and multiple regions of the protein contribute to this interaction^6,13,20^. A recent cryo-electron microscopy study determined the structure of two small TPX2 regions bound to microtubules, which bind to the crest of each protofilament and to the crevasse between adjacent protofilaments^21^. These peptides make up 25 out of 715 amino acid residues and are located within the N-terminal half of the protein, whereas the rest of TPX2 could not be reconstructed. Independent of this region, TPX2’s C-terminal half can autonomously bind to MTs at higher protein concentrations, and significant binding requires at least two of the five alpha-helical domains α3 to α7^13^.

Most recently, it was discovered that TPX2 forms a co-condensate with tubulin directly on the MT^22^. This co-condensation enhances the reaction kinetics of branching MT nucleation in a physiological environment, and is driven by TPX2’s N-terminal half, which is predicted to be mostly disordered (amino acids 1–477). TPX2 behaves like a liquid on the MT, which upon uniformly coating the MT, beads up into droplets that are regularly spaced apart, according to the Raleigh Instability of fluids^23^.

Despite these recent advances in revealing the role of TPX2 and its interaction partners during spindle assembly, the TPX2 three-dimensional structure and how it performs these functions remained unknown. This is likely due to the intrinsic flexibility of TPX2, which precludes its crystallization and hampers cryo-EM reconstructions that are based on averaging thousands of identical particles. In contrast, NMR spectroscopy has advanced structural understanding of several intrinsically disordered proteins (IDPs) in solution, including proteins comprising stress granules^24–26^, the Alzheimer Disease-implicated protein Tau^27,28^ and elastin-like polypeptides^29^. Intrinsically disordered proteins have also been studied in condensed/assembled states by solid-state magic-angle spinning NMR spectroscopy (MAS NMR). Examples include α-synuclein^30^, a key player in Parkinson and Alzheimer’s diseases, as well as phase-separated proteins, such as Edc3, a central hub of mRNA processing bodies^31^, the low-complexity domain in the FUS RNA-binding protein^32^, and the heterochromatin protein HP1α^33^. Despite successful investigations of smaller proteins and protein assemblies, structural characterization of large complexes involving IDPs, such as LLPS proteins assembled on cytoskeletal filaments, represents a challenge. In addition, the rules that govern protein condensation on a molecular surface are not known.

Here, we determined the structure of TPX2 C-terminal minimal active domain (TPX2^α5-α7^) bound to MTs by MAS NMR spectroscopy and molecular modeling and delineated the TPX2^α5-α7^-microtubule interactions. Remarkably, TPX2^α5-α7^ assembles into condensates on the MT lattice, where the protein has rigid and folded regions flanked by intrinsically disordered stretches, which are responsible for the protein’s transient, dynamic binding modes. We compared this structure with its condensate and monodisperse form in solution and demonstrate that the protein is intrinsically disordered in solution and undergoes a structural transition from unfolded to folded states upon binding to MTs. Aromatic and hydrophobic residues were identified to be critical for forming long-range contacts that mediate the structural and dynamic behavior of TPX2^α5-α7^ as a condensate on the MT lattice. The current study represents the atomically detailed characterization of a protein involved in MT-bound condensates and demonstrates the potential of MAS NMR for structural characterization of intrinsically disordered proteins bound to microtubules, and generally, of phase-separated proteins in solid-state biomolecular condensates.

## Results

### The minimal active domain of TPX2 binds microtubules and undergoes liquid–liquid phase separation

**Fig. 1:**
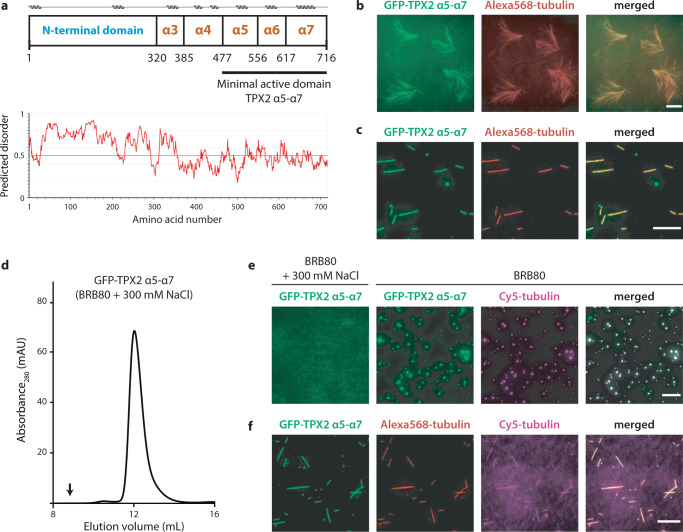
Functional characterization of TPX2^α5-α7^. **a** Domain organization of *Xenopus laevis* TPX2 with predicted alpha-helical regions and predicted intrinsic disorder. **b** Fluorescent images of GFP-tagged TPX2^α5-α7^ (1 µM) (green) co-localization with branched microtubule networks (red) in *Xenopus* egg extract. Data representative of three experimental replicates. **c** Fluorescent images of GFP-tagged TPX2^α5-α7^ (1 µM) (green) binding to GMPCPP-stabilized microtubules (red). Representative of three experimental replicates. **d** Gel filtration elution profile of monodispersed TPX2^α5-α7^ in the presence of 300 mM NaCl. **e** Fluorescent images of monodispersed GFP-tagged TPX2^α5-α7^ (5 µM) (green) with 300 mM NaCl, and in its co-condensed form with soluble tubulin (magenta) in BRB80 buffer alone. Experiments were repeated at least three times. **f** Fluorescent images of GFP-tagged TPX2^α5-α7^ (1 µM) (green) binding to GMPCPP-stabilized microtubules (red) in the presence of additional soluble tubulin (1 µM) (magenta). All scale bars, 10 µm. Experiments were repeated three times with similar results.

We set out to determine how the C-terminal region of TPX2, which contains the domains α5-α7 that are essential for its activity (Fig. 1a, amino acids 477–716), binds to MTs, a prerequisite to elicit branching MT nucleation^34,35^. As shown in Fig. 1b, c, GFP-tagged TPX2^α5-α7^ co-localizes with branched microtubule networks in *Xenopus* egg extract and binds to pre-formed GMPCPP-stabilized microtubules in vitro, consistent with previous results. Notably, TPX2^α5-α7^ does not contain the MT-binding region that was defined in a recent cryo-EM study^21^, indicating that additional MT-binding motifs on TPX2 are present within this minimal active construct.

Full-length TPX2’s ability to form a co-condensate with soluble tubulin enhances the kinetics of branching MT nucleation^22^. We tested whether the minimal active C-terminal region of TPX2 is also able to undergo a LLPS on its own and co-condense with tubulin. To do so, we purified GFP-TPX2^α5-α7^ in a monodispersed state in the presence of 300 mM NaCl (Fig. 1d) and diluted the solution with a buffer that contained lower salt concentrations and Cy5-tubulin. As shown in Fig. 1e, GFP-TPX2^α5-α7^ formed a co-condensate with tubulin, albeit at higher concentrations than the full-length protein.

In the presence of MTs, TPX2 does not undergo liquid–liquid phase separation in solution. Instead, the protein preferentially associates with MTs and recruits soluble tubulin to form a co-condensate on MTs^22^. To determine whether TPX2^α5-α7^ retains this property, we incubated the protein with GMPCPP-stabilized MTs and added Cy5-tubulin. Indeed, Cy5-labeled tubulin bound specifically to MTs only in the presence of GFP-TPX2^α5-α7^ (Fig. 1f).

Taken together, these results show that TPX2^α5-α7^, which contains essential motifs to stimulate branching MT nucleation, retains the key properties of full-length TPX2, namely its co-condensation with tubulin upon binding to the MT lattice. These findings motivated us to investigate how a condensate forms on a molecular surface, by determining the structure of TPX2^α5-α7^ monodisperse in solution, as a condensate in solution, and finally, condensed on MTs.

### TPX2^α5-α7^ is intrinsically disordered in its monodispersed state and as a condensate in solution

**Fig. 2:**
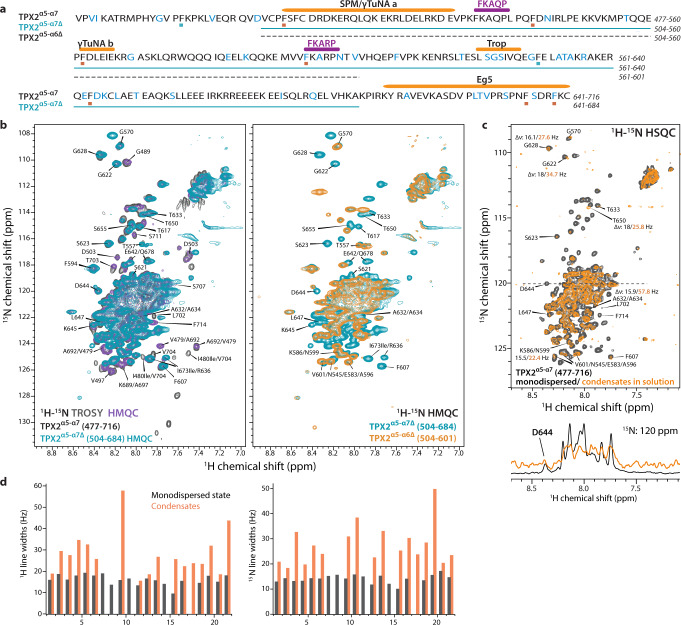
Solution NMR characterization of free TPX2^α5-α7^ in monodispersed solution and condensates. **a** Primary sequence of TPX2^α5-α7^ (residues 477–716), TPX2^α5-α7Δ^, and TPX2^α5-α6Δ^. Assigned residues are shown in cyan. Functional regions are indicated above the sequence. Phe residues where single-site mutations severely affect TPX2 activity in stimulating branching MT nucleation and those that have no effect are marked by red and green dots, respectively. **b** 2D ^1^H-^15^N HMQC solution NMR spectra of U-[^13^C,^15^N]-TPX2^α5-α7^ (purple), U-[^13^C,^15^N]-TPX2^α5-α7Δ^ (cyan), and U-[^13^C,^15^N]-TPX2^α5-α6Δ^ (orange) in monodispersed solution. Left: Overlay of the HMQC spectrum of TPX2^α5-α7Δ^ with the HMQC (purple) and TROSY spectra (gray) of TPX2^α5-α7^. Right: Overlay of the HMQC spectra of TPX2^α5-α6Δ^ and TPX2^α5-α7Δ^. **c** 2D ^1^H-^15^N HSQC spectra of TPX2^α5-α7^ in monodispersed solution (black) and liquid droplets (orange). Representative 1D ^1^H slices are extracted from each 2D HSQC spectrum at the same ^15^N chemical shift. Notable line broadenings for most signals of TPX2^α5-α7^ in the condensed form indicate the formation of larger molecular weight droplets upon LLPS. **d** Comparison of the ^1^H and ^15^N full width at half maximum (FWHM) of representative correlations in the 2D ^1^H-^15^N correlation spectra of TPX2^α5-α7^ in monodispersed solution (black) and liquid droplets (orange). The HMQC spectra were acquired at 18.8 T; the TROSY and HSQC spectra of TPX2^α5-α7^- at 14.1 T. See Supplementary Fig. 1 and Supplementary Table 1 for details.

The structure of monodispersed TPX2^α5-α7^ was characterized by solution NMR. The dispersed TPX2^α5-α7^ protein is intrinsically disordered as evidenced by the small ^1^H chemical shift dispersion in the 2D ^1^H-^15^N HMQC spectrum (Fig. 2b). Nearly 150 resolved cross peaks were detected out of the total of 240 residues. The smaller than expected number of peaks is the consequence of either resonance overlap or chemical exchange giving rise to line broadening. To better assign the IDRs, we also used truncated proteins TPX2^α5-α7Δ^, which is depleted of the N-terminal peptide before α5 and the C-terminal peptide after α7, as well as TPX2^α5-α6Δ^, which is in addition lacking the α7 helix (Fig. 2a). Similar behavior is also observed in the spectra of TPX2^α5-α7Δ^ and TPX2^α5-α6Δ^ (Fig. 2b). Only the terminal residues near the truncated positions exhibit chemical shift perturbations (CSPs) in TPX2^α5-α7Δ^ and TPX2^α5-α6Δ^ with respect to TPX2^α5-α7^, indicating minor structural changes associated with truncation. These results suggest that both shorter constructs retain the structural disorder of TPX2^α5-α7^.

We additionally characterized the condensates of TPX2^α5-α7^ in solution, formed via LLPS at low salt concentration of ~40 mM NaCl. The HSQC spectra indicate that the intrinsic disorder of TPX2^α5-α7^ persists in the condensate, as is evident from the small chemical shift dispersion, which is similar to that observed for the monodisperse TPX2^α5-α7^ (Fig. 2c). Furthermore, given a very limited number of small CSPs, there are no major structural changes occurring upon the transition of TPX2^α5-α7^ from soluble monodisperse to the condensed phase. The most pronounced difference between the two forms is the line broadening observed for most residues in the condensate, as shown in Fig. 2d, Supplementary Fig. 1, and summarized in Supplementary Table 1. This broadening is a characteristic of intermediate-regime conformational exchange and likely associated with the transient formation of large molecular weight droplets upon LLPS, which have slower molecular tumbling and higher rigidity than the dispersed protein. This observation is consistent with the formation of TPX2^α5-α7^ liquid droplets observed by fluorescence microscopy (Fig. 1e) and the rapid loss of dynamics associated with aging of the TPX2^α5-α7^ condensates as has been reported^22^. Interestingly, individual residues exhibit varying degrees of line broadening, indicating that LLPS induces different changes in local mobility throughout the protein (Supplementary Fig. 1 and Supplementary Table 1). One notable example is the segment spanning G622 to A634, which exhibits higher degree of rigidity in the TPX2^α5-α7^ condensates, as evidenced by significantly higher-than-average degree of line broadening for G622, G628, T633, and A632/634.

### TPX2^α5-α7^ bound to microtubules is rigid and possesses static disorder

**Fig. 3:**
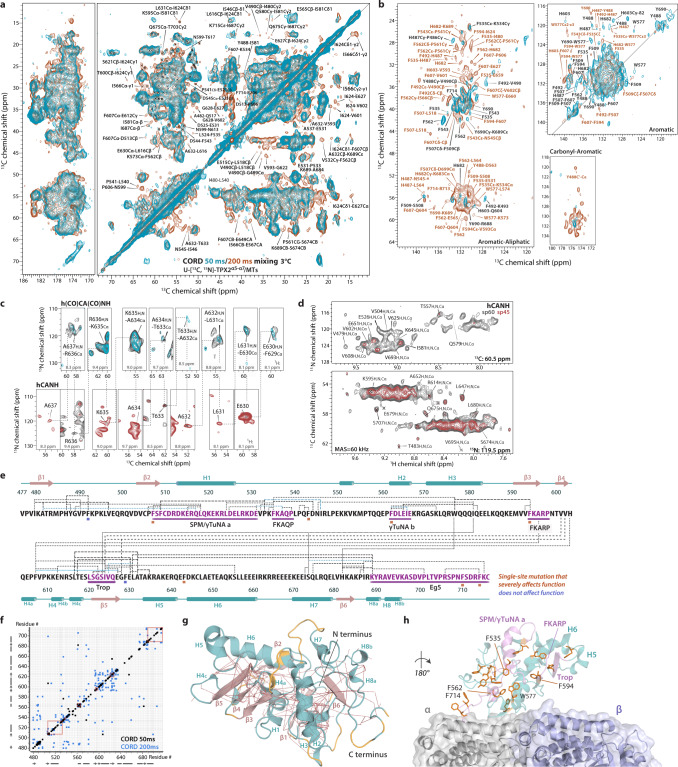
MAS NMR spectra and 3D structure of TPX2^α5-α7^ assembled with microtubules (MTs). **a**, **b** 2D ^13^C-^13^C CORD spectra of fully protonated TPX2^α5-α7^/MT assemblies acquired with mixing times of 50 ms (cyan) and 200 ms (brown). Long-range inter-residue correlations detected with a CORD mixing time of 200 ms are labeled in brown. Representative correlations involving aromatic sidechains in the 50 ms CORD spectrum are labeled in black. The spectra were acquired at 14.1 T, the sample temperature was 3 °C, and the MAS frequency was 14 kHz. **c** Representative backbone walk (segment F629-A637) showing sequential resonance assignments of TPX2^α5-α7^, using the 3D ^1^H-detected hCANH and h(CO)CA(CO)NH spectra of deuterated U-[^13^C,^15^N]-TPX2^α5-α7^/MT assemblies. **d** Representative 2D ^15^N-^1^H and ^13^C-^1^H planes of 3D hCANH spectra. ^1^H-detected MAS NMR spectra were acquired at 20.0 T; the MAS frequency was 60 kHz. **e** Long-range contacts mapped on the TPX2^α5-α7^ sequence. Inter-residue contacts identified in the CORD spectra are shown as dot-dashed lines. Secondary structures derived from chemical shift index are indicated; cylinders and arrows represent helices and β-sheets, respectively. **f** Interatomic contact map. The contacts observed in the CORD spectra with mixing times of 50 ms (black) and 200 ms (blue) are shown. Contacts in the functional regions are marked by orange rectangles. **g** Molecular structure of TPX2^α5-α7^ bound to MTs determined by MAS NMR. Experimental distance restraints were mapped as red dashed lines. Unassigned residues are shown in yellow. Helices and β-sheets are shown in cyan and salmon, respectively. **h** Structural arrangement of aromatic residues in TPX2^α5-α7^ (light cyan) bound to MTs at the tubulin interdimer interface (α tubulin, gray; β tubulin, light purple).

We next characterized TPX2^α5-α7^ bound to polymerized MTs by MAS NMR spectroscopy to obtain atomic-level structural information. From a combination of 10 2D and 3D ^13^C- and ^1^H-detected correlation spectra acquired on two protonated or deuterated TPX2^α5-α7^/MT samples, we derived backbone and side chain chemical shift assignments for 195 residues of TPX2^α5-α7^ (Fig. 3a–d, Supplementary Figs. 2–4). Residue-specific backbone assignments were inferred from the 3D ^1^H-detected hCANH and h(CO)CA(CO)NH spectra, and a representative sequential backbone walk for segment F629–637 is shown in Fig. 3c. Most correlations for TPX2^α5-α7^ residues are present in the dipolar-based ^13^C- and ^1^H-detected 2D and 3D spectra. Given the small amount of isotopically labeled protein bound to MTs that do not contain isotopic labels (and are, therefore, invisible in the ^13^C-based NMR correlation spectra), the surprisingly high signal-to-noise ratio of these spectra suggests that remarkably, TPX2^α5-α7^ is overall rigid when bound to MTs. At the same time, there is substantial degree of static conformational disorder in MT-bound TPX2^α5-α7^. The 2D ^13^C-^13^C combined R2_n_^ν^ driven spin diffusion (CORD)^36^ and ^13^C-^1^H heteronuclear correlation (HETCOR) spectra (Fig. 3a and Supplementary Fig. 5, respectively) contain regions with well-resolved cross peaks as well as those exhibiting spectral overlap, which correspond to residues in the structured and statically disordered regions, respectively. An important feature of the ^1^H-detected spectra is considerable site-to-site variation in the ^1^H line widths (Fig. 3d), indicative of static conformational heterogeneity of TPX2^α5-α7^ on MTs. Interestingly, certain regions show multiple sets of ^1^H peaks associated with discrete conformers for Arg, Gln, Glu, and Lys residues, and likely spanning Glu/Gln-rich regions. While at present we cannot assign these multiple sets of peaks to specific local environments, we hypothesize that these correspond to TPX2^α5-α7^ molecules bound to MTs in a condensed phase rather than a monodisperse form, given that the TPX2^α5-α7^ concentration used for preparing TPX2^α5-α7^/MT assemblies is 1 mM, much higher than its critical concentration for droplet formation 5–10 μM. This hypothesis is consistent with the fact that full-length TPX2 forms condensates that coalesce after uniformly coating MTs^22,23^, the process driven by transient intermolecular interactions. Since TPX2^α5-α7^ shares the ability to undergo LLPS, its binding to MTs may occur in a similar manner.

Importantly, we determined the secondary structure of TPX2^α5-α7^ bound to MTs from the ^13^C chemical shifts^37^. The TPX2 secondary structure is unavailable by any other techniques due to its intrinsically disordered nature and hence desirable. As shown in Fig. 3e and Supplementary Figs. 6, 7, the α-helical regions comprise stretches of residues D513-E526, F562-E567, A571-E583, Q604-E618, A634-Q641, D644-I661, K670-L680, and R688-D699 (sequentially named helix 1–8, or H1–8). The edge of each, α5, α6, and α7 helix is clearly delineated from the experimental data, and the chemical shifts also reveal two previously unknown α-helical regions, Q604-E618 (H4) and R688-D699 (H8). In contrast, only half of α6 is predicted to be α-helical by the sequence-based Jpred^38,39^ algorithm. Important to note, the H8 helix at the C-terminus comprises half of the functional region critical for the interaction between TPX2 and kinesin Eg5. Furthermore, the major segments in the two regions that share homology with γ-TuRC-mediated nucleation activator (γTuNA) motifs are in helices, such as H1 helix located in the middle of the first functional region γTuNAa and H2 helix that comprises γTuNAb region. The MAS NMR data also reveal six stretches of residues possessing β-sheet structures (β1–6) located in multiple functional regions. These include the M591-V602 segment (β3 and β4) that incorporates the FKARP motif, which occurs several times in TPX2 and could be important for microtubule nucleation^13^, and the S621-E627 segment (β5) that overlaps with the region missing in *Xenopus tropicalis* TPX2 (Trop), which negatively regulates microtubule nucleation^40^.

### Long-range contacts and tertiary structure of TPX2^α5-α7^ bound to microtubules

The experimentally determined structure of TPX2^α5-α7^ was unavailable prior to this study. The sequence-based structure predictions by AlphaFold 2^41,42^ (AF2) and Robetta^43^ generate unfolded TPX2^α5-α7^ models and reveal three major helices that were previously predicted by Jpred (Supplementary Fig. 8). Unlike many protein structures that are successfully predicted by AF2 with high confidence, TPX2 represents one of the proteins that are poorly predicted, with low accuracy due to the presence of intrinsically disordered regions. Therefore, it is not surprising that AF2 fails to predict an accurate conformation and provide additional structural information for TPX2.

To assess the tertiary structure of TPX2^α5-α7^ bound to microtubules, we recorded CORD spectra with a longer mixing time of 200 ms and detected medium- and long-range contacts (Fig. 3a, b and Supplementary Table 5). Non-trivial contacts are identified for numerous residues indicative of multiple segments with folded structures. In total, we unambiguously assigned 163 unique non-redundant inter-residue contacts (Supplementary Table 6). The long-range contacts in the aliphatic region mapped on the primary sequence and the 2D contact map are shown in Fig. 3e, f. It is clear from the contact map that a large fraction of long-range correlations corresponds to residues in the functional regions, indicating that these have well-defined structure. Interestingly, numerous long-range correlations involving the aromatic Phe residues were observed, including the contacts between the Phe residues which are evenly distributed in the sequence, suggesting their function as ‘stickers’ between ‘spacers’^44^. Importantly, single-site mutations in these residues were found to severely affect the function of TPX2. We therefore posit that Phe residues may be important for self-association or/and phase separation behavior of TPX2^α5-α7^. Specifically, Phe residues form contacts with adjacent residues, including Arg, Gln, Glu, and Asn. These amino acids possess side chains containing pi-orbitals that can form pi-pi contacts^45^. The identification of over 54 inter-residue contacts associated with hydrophobic Phe residues (Fig. 3b) suggests that these aromatics facilitate the tertiary fold of TPX2^α5-α7^ molecule and the formation of condensates on MTs.

In addition to the above findings, we observed several inter-residue contacts involving charged residues. Interestingly, the majority of these contacts are between charged and hydrophobic residues. We also identified numerous medium- and long-range inter-sticker contacts between hydrophobic residues, especially within the Trop region and between the residues comprising the FKARP motif and the Trop region (Fig. 3e). For instance, multiple correlations between the I624 in the Trop region and the adjacent valine residues were observed. These hydrophobic interactions may play a critical role in regulating the condensation of the folded TPX2^α5-α7^ molecules upon binding to MTs.

**Fig. 4:**
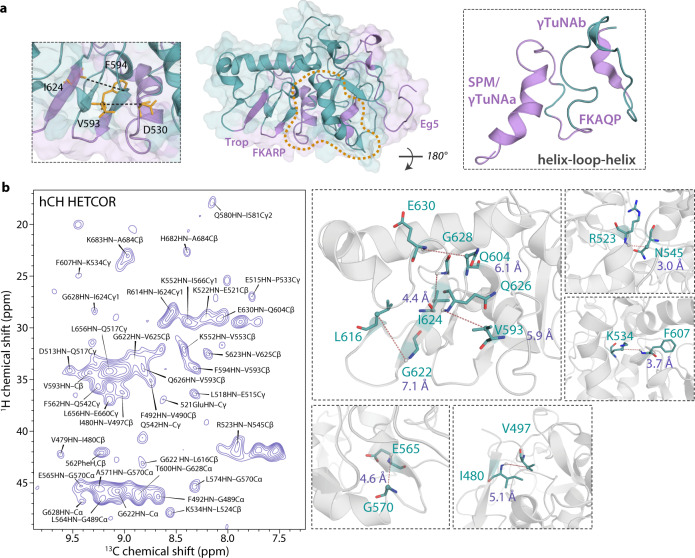
Structure of the functional regions in TPX2^α5-α7^ bound to microtubules. **a** Conformation of the functional regions and regions of interest (purple) in TPX2^α5-α7^, constrained by experimental ^13^C-^13^C restraints. The γ-TuNA a and b motifs and the loop containing FKAQP motif form a helix-loop-helix structure. **b** Medium and long-range intramolecular ^13^C-^1^H restraints in TPX2^α5-α7^ on MTs, revealed by 2D CH heteronuclear correlated (HETCOR) MAS NMR spectrum. A number of intra and inter-residue contacts between amide ^1^H and backbone or sidechain ^13^C atoms were identified. The inter-residue ^13^C-^1^H restraints correspond to typical interatomic distances of 3–8 Å in the MAS NMR structure of TPX2^α5-α7^ bound to MTs. Representative ^13^C-^1^H distances in the functional motifs and loop regions are shown; the corresponding residues and atoms are shown in sticks and spheres, respectively. The spectrum was acquired on deuterated TPX2^α5-α7^/MT assemblies at a magnetic field of 20.0 T and a fast MAS frequency of 60 kHz; long cross-polarization contact times of 2.7–2.8 ms were used.

To better understand the structure-function relationship of TPX2^α5-α7^ bound to MTs, we determined the structural model of TPX2^α5-α7^ based on the MAS NMR experimental restraints (Fig. 3g, Supplementary Figs. 9, 10a). In total 342 ^13^C-^13^C distance restraints obtained from the homonuclear correlation spectra and 288 dihedral angle restraints were used (Supplementary Table 6). As is shown in Fig. 3g, the folded TPX2^α5-α7^ structure is mainly defined by medium and long-range interatomic distance restraints. Most segments with assigned chemical shifts and distance restraints, including the functional regions, are defined with high confidence, while most of the residues for which experimental restraints are scarce or lacking comprise the loops. In our MAS NMR structure, the TPX2^α5-α7^ protein adopts an overall elongated structure with H1 and H4 folded inward. All functional regions and regions of interest including FKARP and FKAQP are located in the periphery of TPX2^α5-α7^ structure and exposed for interactions with essential binding partners (Fig. 4a, Supplementary Fig. 10b, c). The helices of γTuNA a and b motifs are connected by the loop containing FKAQP, thus forming a helix-loop-helix structure (Fig. 4a). This finding is consistent with the cryo-EM structure of γTuNA from human CDK5Rap2 bound to γTuRC, where the γTuNA peptide is proposed to be a helix-loop-helix motif^46^. The sidechain conformations of these functional motifs and the loop regions in TPX2^α5−α7^ are also validated by the medium and long-range inter-residue ^13^C-^1^H restraints (Fig. 4b, Supplementary Fig. 11). These contacts were extracted from the 2D CH HETCOR spectra of deuterated TPX2^α5−α7^/MTs acquired with longer contact time and correspond to typical interatomic distances of 3–8 Å in the 3D structure of TPX2^α5−α7^ bound to MTs. Furthermore, although the hydrophobic residues are distributed randomly in the sequence, the aromatic residues are clustered and form dense interactions in the core of the folded TPX2^α5-α7^ molecule (Fig. 3h, Supplementary Fig. 10d). The side chains of the aromatic residues are oriented for tight packing, which stabilize the TPX2^α^^5-^^α^^7^ folding. These arrangements likely promote the interactions between hydrophobic residues despite their random distribution in the sequence and tertiary structure, and are consistent with aromatic residues acting as stickers for LLPS of TPX2^α5-α7^. These findings highlight that TPX2^α5-α7^ is a LLPS-prone protein, as conserved hydrophobic cores are generally required for well-structured proteins^47^. As discussed above, the Phe residues play important roles in the cellular functions of TPX2 protein and the single-site mutations have a detrimental effect on TPX2’s ability to stimulate branching MT nucleation. Since MT branches were shown to nucleate from phase-separated TPX2 droplets on microtubules^23^, our results suggest that mutations of the key Phe residues may interrupt these dense aromatic interactions affecting the TPX2 folding and formation of TPX2 droplets on MTs. As a result, the single-site mutations lead to loss of function of TPX2 as MT branches cannot nucleate efficiently in the absence of TPX2 condensates.

The structural model of TPX2^α5-α7^ derived from MAS NMR provides a first description of a protein that is involved in a condensate on MTs. As TPX2^α5-α7^ is disordered in solution, our results suggest that TPX2^α5-α7^ undergoes a structural transition from unfolded to folded states upon binding to microtubules. Intrinsically disordered regions have been shown to drive protein condensation^48–53^, but several studies have also suggested that folded domains or partially folded structures play a role in promoting phase separations through folding upon interacting with binding partners^54–57^. Based on our model, we hypothesize that the folding of TPX2^α5-α7^ protein facilitates its condensation after binding to MTs.

### Intermolecular interfaces of TPX2^α5-α7^ with microtubules

**Fig. 5:**
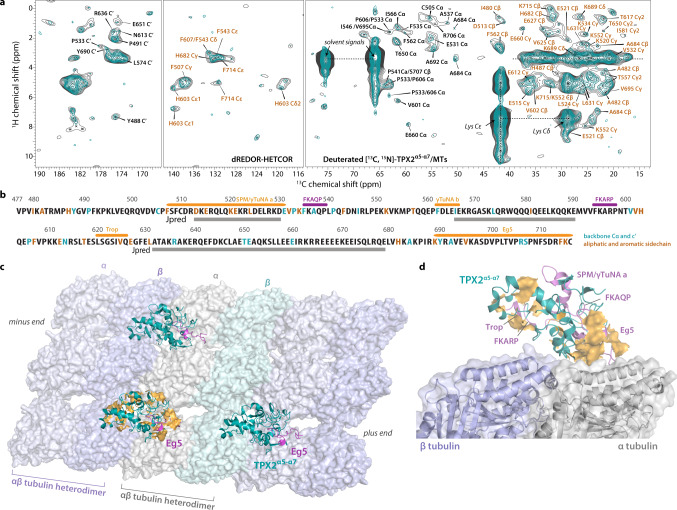
Intermolecular interfaces of TPX2^α5-α7^ with polymerized microtubules. **a** 2D double-REDOR (dREDOR) filtered ^1^H-^13^C HETCOR spectrum of deuterated U-[^13^C,^15^N]-TPX2^α5-α7^/MT assembly. Backbone and side chain correlations are shown in black and orange, respectively. **b** Residues comprising the interface with MTs mapped on the TPX2^α5-α7^ primary sequence. **c** Structural model of TPX2^α5-α7^ bound to MT filaments, generated by blind docking of MAS NMR structure of TPX2^α5-α7^ onto the cryo-EM structure of polymerized MTs (PDB: 3J6G^94^) in ClusPro^91^. TPX2^α5-α7^ (teal) binds to MTs at the intersection between neighboring tubulin heterodimers and at the groove between two adjacent MT protofilaments, with Eg5 region (magenta) remaining exposed. **d** Predominant binding mode of TPX2^α5-α7^ (light teal) on a βα tubulin dimer in microtubule filaments. The structural model was generated from the NMR restraint-guided docking in HADDOCK^92^. The residues comprising the binding interface are colored orange. The functional regions and FKARP/FKAQP segments are colored light magenta. The γ-TuNA a and b motifs are positioned facing away from the MT, which allows their direct interactions with the 2.2 MDa γ-TuRC and recruit it for MT nucleation.

To understand the structural basis for TPX2^α5-α7^ behavior on the MT lattice and reveal the intermolecular interfaces of TPX2^α5-α7^ with microtubules, we performed double rotational echo double resonance (dREDOR) filter-based experiments^58,59^. All the Cα signals and majority of Cβ signals of TPX2^α5−α7^ are absent in the 1D dREDOR-based spectra, indicating that the amide proton magnetizations of deuterated TPX2^α5−α7^ were dephased by the dipolar filters. A limited number of intermolecular correlations arising from the MTs were observed in dREDOR ^1^H-^13^C spectra (Fig. 5a, Supplementary Fig. 12). Of these, there are unique correlations between the protons in MTs and the backbone/sidechain ^13^C atoms corresponding to multiple residues in TPX2^α5-α7^ (shown in Fig. 5a, b and summarized in Supplementary Table 7). These residues either comprise the interface of TPX2^α5-α7^ with MTs or are in proximity to MT protofilaments.

The structural models derived from molecular docking reveal the dominant binding mode of TPX2^α5-α7^ with MTs. The blind docking of MAS NMR structure of TPX2^α5-α7^ onto the cryo-EM structure of polymerized MTs predicts that H3 and β3-5 segments form the binding interface with MTs (Fig. 5c). Remarkably, this structural model is fully consistent with the MAS NMR results discussed above: the same TPX2 residues comprise the binding interface of TPX2^α5-α7^ with MTs in both dREDOR-based experiments and the docking calculations. Furthermore, the dominant binding mode is also predicted by the experimental restraint-guided docking of the TPX2^α5-α7^ structure on a βα tubulin inter-heterodimer (Fig. 5d). The minor binding mode predicted by both blind and data-guided dockings was ruled out since it involves the Eg5-binding region as the primary interacting region with microtubules (Supplementary Fig. 13), thus contradicting prior experimental finding that the removal of Eg5 domain at the C-terminus does not significantly reduce its binding affinity with MTs^13,60^.

In this structural model, TPX2^α5-α7^ binds to MTs at the intersection between two neighboring tubulin heterodimers and at the groove between two adjacent MT protofilaments (Fig. 5c, Supplementary Fig. 10e). TPX2^α5-α7^ molecules interact with MTs between the β tubulin of one heterodimer and the α tubulin of the succeeding heterodimer, indicating that the protein promotes the assembly of tubulin dimers and protofilaments. This binding model is in accord with TPX2’s role in stabilizing MTs^13,61,62^, and promoting tubulin polymerization at the MT plus end^61^. It also agrees with our biochemical studies showing that TPX2^α5-α7^ recruits tubulin. The tubulin residues in MTs with tentatively assigned ^1^H resonances based on the SHIFTX2^63^ predicted chemical shifts are consistent with those that interact with TPX2^α5-α7^, which extensively support this TPX2^α5-α7^-MT-binding mode (Supplementary Fig. 14 and Supplementary Table 8). The overall position of TPX2^α5-α7^ on MTs also agrees with the cryo-EM density of its two N-terminal segments on the MT lattice^21^, showing that the TPX2 central region (residues 274–659) decorates MTs by positioning itself between the MT protofilaments and between αβ tubulin heterodimers. The C-terminus Eg5-binding region remains exposed, which ensures its accessibility to Eg5. These interactions are critical for targeting Eg5 motors to the spindle and promoting bipolar spindle assembly as well as inhibitions of Eg5 processivity on MTs. Moreover, only three positively charged residues in TPX2^α5-α7^ interact with MTs according to the dREDOR experiments, corroborating the previous reports that the negatively charged E-hook is not necessary for MT binding^60^. In addition, our structural model reveals that the SPM/γTuNAa and FKAQP regions are projected away from MTs for predicted interactions with the universal nucleator γ-TuRC, while other functional regions are arranged for possible minor contacts with MTs (Fig. 5d, Supplementary Fig. 10c). These results lay the foundation for understanding how C-terminal domain of TPX2 activates γ-TuRC and inhibit Eg5 activities through the direct interactions. Finally, we note that in the docking model, TPX2^α5-α7^ molecules only sparsely decorate MTs at specific positions, yet form a condensate on MTs, which is consistent with AFM measurements that estimate the number of TPX2 molecules in the condensates^23^. The molecular orientation of TPX2^α5-α7^ on MTs is not experimentally determined and we speculate that the protein might adopt slightly different orientation from the model derived herein.

### Dynamic residues in TPX2^α5-α7^ bound to the microtubule lattice

**Fig. 6:**
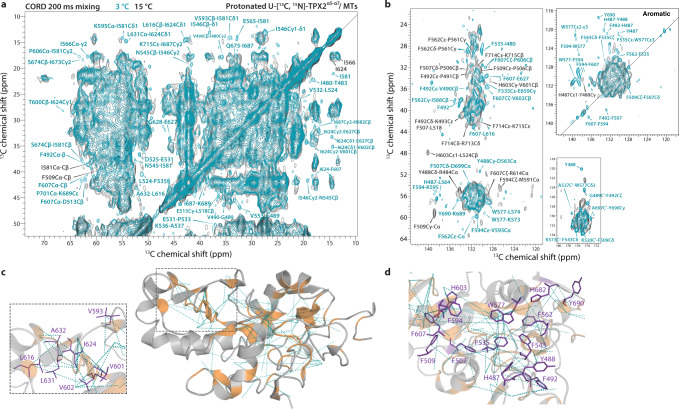
Dynamic residues in TPX2^α5-α7^ bound to MTs. **a**, **b** Representative aliphatic (**a**) and aromatic (**b**) regions of the 2D ^13^C-^13^C CORD spectra of U-[^13^C,^15^N]-TPX2^α5-α7^/MTs, acquired with 200 ms mixing time at 3 °C (teal) and 15 °C (gray). Representative intra- and inter-residue correlations observed exclusively at 3 °C are colored teal. The contacts present at 15 °C are labeled in black. The spectra were acquired at 14.1 T; the MAS frequency was 14 kHz. **c** The contacts involving dynamic residues mapped onto the structure of TPX2^α5-α7^. Contacts that are absent at 15 °C are shown as teal dashed lines; the corresponding dynamic residues are colored in orange. The zoom-in view shows representative hydrophobic residues that are more dynamic at higher temperature. **d** Expanded view of the TPX2^α5-α7^ structure showing the aromatic residues (purple) in the core. The cross peaks for these residues are absent at 15 °C suggesting greater mobility at higher temperature and different/heterogeneous sidechain orientations.

To understand the dynamic behavior of TPX2, we probed the conformational flexibility of TPX2^α5-α7^ bound to MTs by recording temperature-dependent spectra. The 2D ^13^C-^13^C correlation spectra with a mixing time of 200 ms were acquired at estimated sample temperatures of 3 and 15 °C, the latter being in the range of *Xenopus laevis’* physiological temperatures (Fig. 6a, Supplementary Fig. 15b). While no major structural changes of TPX2^α5-α7^ were observed, as is evident from similar spectral resolution and no significant chemical shift perturbations for majority of the signals between the two temperatures, there are pronounced differences in the dynamic behavior associated with signal intensity changes of specific residues. First, a number of correlations in both aliphatic and aromatic regions are absent at 15 °C (Fig. 6a, b), indicating that the corresponding residues are more dynamic at the higher temperature. Not surprisingly, most of these residues comprise loops or disordered regions (Fig. 6c), including the N- and C-terminus. Specifically, a few of the missing signals are associated with terminal residues 480–492 and 713–715. Several correlations corresponding to Lys side chains are also absent as these are typically more flexible than other types of side chains. Interestingly, the signal intensities of backbone atoms for many residues are also affected, indicating their temperature-dependent mobility. The corresponding sites are located in the β1-β2 loop, H1, H2, β3, β4-H4, and β5-H6 regions (Fig. 6c, Supplementary Fig. 15a, c) and involve all the functional regions, including the SPM/γTuNAa, γTuNAb, Trop, the Eg5-binding domain, and FKARP/FKAQP segments.

The dynamic residues detected by MAS NMR are primarily hydrophobic, including aromatic residues (Phe, Tyr, and Trp), a few negatively charged residues (e.g., D525, E627, and D699), and polar residues (e.g., N545) (Fig. 6c, d). The sidechains of several aromatic residues, e.g., F492, F507, F543, F562, W577, F594, F607, and Y690, exhibit greater mobility at higher temperature and may adopt different sidechain orientations, as evidenced by new correlations arising for F492, F507, F607, and F594 (Fig. 6d). This observation appears to be significant given that these aromatics form dense interactions in the core and stabilize TPX2^α5-α7^ folding. On the basis of these results we propose that the condensates on MTs have higher dynamicity and TPX2^α5-α7^ undergoes local changes in backbone and side chain dynamics in its physiological environment as a response to the temperature change, which in turn affects the inter-residue interactions between the aromatics and gives rise to the conformational changes in these “key stickers” triggering TPX2^α5-α7^ condensation.

## Discussion

Using comprehensive MAS NMR experiments in combination with protein structure modeling, we described the three-dimensional molecular structure of the TPX2 minimal active domain in its monodisperse state, as a condensate in solution, and as a condensate coating the MT lattice, in addition to defining its binding modes with MTs. This structural comparison provides unique insight into the structure transition of condensed proteins on molecular surfaces and helps explain how TPX2 forms the basis of a MT branch site during spindle assembly.

Surprisingly, the TPX2 minimal active domain has similar structural disorder in its monodisperse form and when condensed in solution. The major difference is that intermediate-regime conformational changes as well as changes in local mobility of individual residues occur in the solution condensate form, indicating its LLPS. Whereas methods like X-ray crystallography or Cryo-EM cannot capture the structure of an IDR-based protein on a MT, we show here that the combination of sophisticated MAS NMR experiments and molecular modeling can fill this void. Our results reveal that the elongated TPX2^α5-α7^ molecule, being rigid and displaying static disorder, binds to MTs between two adjacent protofilaments and at the tubulin interdimer cross section. Structural arrangements of the aromatic residues and their involvement in long-range contacts, together with temperature-induced dynamic rearrangements, suggest that these residues are essential for the tertiary fold of TPX2^α5-α7^ and formation of the condensates on MTs. This is consistent with a previous study of prion-like domains (PLDs) that proposed a “stickers and spacers” model where aromatic residues serve as “stickers” for phase separation and are uniformly flanked by disordered “spacers”^44^. Similarly, several aromatic residues were found to be key factors for the LLPS of the granule protein TDP-43^64^.

TPX2 is essential in branching MT nucleation, a major pathway to form spindles in *Xenopus* and human cells^6,8,9^. Yet, how it achieves this function remains to be uncovered. Our findings reveal the binding mode of TPX2^α5-α7^ with the MT and thus illuminate the structural basis of the branch site. This minimal active domain of TPX2 is positioned such that it can form a co-condensate with tubulin and organizes into a condensed phase on MTs. The γ-TuNA a and b motifs, among those encompassing the residues critical for its function, form a helix-loop-helix motif similar to the γ-TuNA domain of the centrosomal CDK5RAP2. This indicates that proteins localizing to different MT organizing centers may harbor the same unique sequence to activate γ-TuRC. Moreover, the γ-TuNA a and b motifs are positioned facing away from the MT, where they can directly interact with the 2.2 MDa γ-TuRC and thus recruit it for MT nucleation. Similarly, the FKARP motif is positioned away from the MT, although its concrete function still needs to be identified. The C-terminal residues involved in Eg5-binding point away from TPX2 and the MT, making it possible for TPX2 to interact with this motor protein for spindle assembly.

Finally, the MAS NMR structure provides important clues regarding the LLPS mechanism and structure-function relationship of TPX2 condensates on the MT lattice. The current study provides atomic-level structural information for a protein that forms a condensate on cytoskeletal filaments. This work lays the foundation for structural and dynamic studies of condensed microtubule-associated proteins on MTs and phase-separated proteins in large biological assemblies.

## Methods

### Chemicals

The ^15^NH_4_Cl, U-^13^C_6_ glucose, U-[^13^C_6_, D_7_]-glucose, and D_2_O (99.8% isotopic purity) were purchased from Cambridge Laboratories, Inc.

### Protein expression and purification

The *Xenopus laevis* TPX2^α5-α7^ (amino acids 477−716) construct was fused with a N-terminal His-GFP tag using the 9GFP (Macrolab) vector and expressed *in E. Coli* Rosetta 2 cells (EMD Millipore) for 16–18 h at 16 °C in Terrific Broth media. Fully protonated and uniformly-[^13^C,^15^N]-labeled TPX2^α5−α7^ was expressed in M9 minimal media containing ^15^NH_4_Cl and U-^13^C_6_ glucose as the sole nitrogen and carbon sources. Deuterated U-[^13^C,^15^N]-TPX2^α5-α7^ protein was expressed in M9 minimal media in 100% D_2_O supplemented with ^15^NH_4_Cl and U-[^13^C_6_, D_7_]-glucose. Cells were first grown at 37 °C in unlabeled M9 minimal media and pelleted by centrifugation at 4500 × *g* for 20 min after the OD^600^ reached 0.7–1.0. The cell pellets were then transferred to M9 minimal media prepared in 70% D_2_O and grew for 7 h at 37 °C. Cells were pelleted and resuspended in 100% D_2_O M9 minimal media for an overnight growth at 37 °C. All the media used for cell growth before the next transfer incorporate ^15^NH_4_Cl and unlabeled glucose as the sole nitrogen and carbon sources. After cells were pelleted and transferred to 2 L 100% D_2_O Terrific media enriched with U-[^13^C_6_, D_7_]-glucose, the overexpression of deuterated U-[^13^C, ^15^N]-enriched TPX2^α5-α7^ was induced with 0.5 mM IPTG at 16 °C for 16–18 h and cells were harvested at 4500 × *g* for 20 min.

Protonated and deuterated U-[^13^C,^15^N]-labeled TPX2^α5-α7^ proteins were purified by the same three-step chromatography. After cells were lysed by sonication, the cell lysate was centrifuged, and resulting supernatant was purified with HisTrap affinity chromatography. The eluted fractions containing TPX2^α5−α7^ protein were combined and further purified by MonoS cation-exchange chromatography. His-GFP tag was removed from TPX2^α5-α7^ protein by a cleavage with TEV protease at a ratio of 1:100 at 4 °C for 18 h. Protein were then purified using the NiNTA (Qiagen) column to remove the His-tagged protease and cleaved GFP. The flowthrough containing purified TPX2^α5−α7^ protein was collected, buffer exchanged in phosphate buffer (25 mM phosphate, 8 mM MgCl_2_, 1 mM DTT, 1 mM EGTA, pH 6.8) and concentrated to ∼1 mM for preparation of NMR samples. All buffers used for protein purifications were prepared in 100% H_2_O. Gel filtration was performed using a Superdex 200 Increase 10/300 GL column to check the aggregation of TPX2^α5−α7^ at 300 mM NaCl.

To attain high-level ^1^H back-substitutions of the deuterium at exchangeable sites through H/D exchange, the deuterated TPX2^α5−α7^ protein solution was back-exchanged against buffer in 100% H_2_O and kept at 4 °C for 2 days. Deuterated TPX2^α5-α7^ was used for preparation of deuterated TPX2^α5−α7^/microtubule assemblies for proton-detected MAS NMR experiments.

Bovine brain tubulin was labeled with Cy5- (Sigma, GEPA 15101) or Alexa-488 NHS ester (Thermo Fisher Scientific, A20100) to 54% labeling or biotin-PEG4-NHS (Thermo Fisher, 21330).

### Fluorescence microscopy

CSF extracts were prepared from *Xenopus laevis* oocytes as described previously^65^. Extract reactions were done in flow chambers prepared between glass slides and 22 × 22 mm, 1.5 coverslips (Fisherbrand, 12-541B) using double-sided tape. In all reactions 75% of the total volume was extract, and 25% was a combination of other components or CSF-XB (100 mM KCl, 10 mM K-HEPES, 1 mM MgCl_2_, 0.1 mM CaCl_2_, 5 mM EGTA, pH 7.7) + 10% w/v sucrose. Reactions were done in the presence of 0.5 mM sodium orthovanadate (NEB, P0758S) to avoid sliding of microtubules on the glass surface, 0.89 µM fluorescently-labeled tubulin, and with 0.5 µM GFP-TPX2^α5−α7^.

Single-cycled GMPCPP-stabilized microtubules were made as previously described^66^. Briefly, 12 μM unlabeled tubulin + 1 μM Alexa-568 tubulin + 1 μM biotin tubulin was polymerized in BRB80 in the presence of 1 mM GMPCPP (Jena Bioscience, NU-405L) for 1 h at 37 °C. GMPCPP-stabilized microtubules were attached to DDS-treated coverslips using a commercial anti-biotin monoclonal antibody (Thermo Fisher, 03-3700) diluted 1:10 in BRB80^66^. Using the same flow chamber setup as above, 1 µM TPX2^α5−α7^ was subsequently added and incubated for 5 min before imaging. To test the recruitment of unpolymerized tubulin by TPX2^α5−α7^, both in solution and on polymerized microtubules, the two proteins were added at equimolar concentrations.

Total internal reflection fluorescence (TIRF) microscopy was performed with a Nikon TiE microscope using a 100 × 1.49 NA objective. Andor Zyla sCMOS camera was used for acquisition, with a field of view of 165.1 × 139.3 µm. 2 × 2 binned, multi-color images were acquired using NIS-Elements software (Nikon). All adjustable imaging parameters (exposure time, laser intensity, and TIRF angle) were kept the same within experiments.

### Sample preparations for solution and MAS NMR spectroscopy

The solution NMR samples of U-[^13^C,^15^N]-TPX2^α5−α7^ in monodispersed solution and condensed form were prepared by diluting the concentrated TPX2^α5−α7^ solution with buffers containing different concentrations of salt. A typical NaCl concentration of 300 mM is used to prevent phase separation of TPX2^α5−α7^ in solution since the TPX2^α5−α7^ condensates form at salt concentrations lower than 50 mM. For preparation of monodisperse TPX2^α5−α7^ solution, 0.15 mL 1.2 mM U-[^13^C,^15^N]-TPX2^α5−α7^ solution (300 mM NaCl) was diluted with phosphate buffer (25 mM sodium phosphate, 8 mM MgCl_2_, 1 mM DTT, 1 mM EGTA, 10% D_2_O, pH 6.8) to a final TPX2^α5−α7^ concentration of 0.4 mM with 100 mM NaCl. The TPX2^α5−α7^ condensates in solution were prepared by dilution of TPX2^α5−α7^ solution in phosphate buffer depleted of NaCl. The final TPX2^α5−α7^ concentration in its condensates was 0.16 mM (in 0.45 mL phosphate buffer, 40 mM NaCl, 10% D_2_O, pH 6.8). The amounts of TPX2^α5−α7^ molecules in the monodisperse solution and condensed form are ca. 0.18 μmol and 0.072 μmol, respectively. The optimal salt concentration was determined by characterization of TPX2^α5−α7^ liquid droplet formation using TIRF microscopy. The solution NMR samples of truncated TPX2^α5−α7^ constructs were prepared using the same protocol as the full-length protein.

### Preparation of polymerized MTs and TPX2^α5−α7^/MT assemblies

The paclitaxel-stabilized microtubules were polymerized from bovine brain tubulin in BRB80 (80 mM PIPES, 1 mM MgCl_2_, and 1 mM EGTA, pH 6.8) (PurSolutions). Tubulin at 25 μM was polymerized in BRB80 supplemented with 1 mM GTP at 37 °C for 20 min. The polymerized microtubules were stabilized with 50 μM taxol and centrifuged at 270,000 × *g* (TLA-100) for 15 min. The resulting microtubule pellets were resuspended in concentrated protonated or deuterated TPX2^α5−α7^ solution containing 40 μM taxol in 25 mM phosphate buffer. The mixtures of TPX2^α5−α7^ and microtubules were incubated at room temperature for 45 min and centrifuged through a 40% glycerol-BRB80 cushion at 270,000 × *g* for 15 min at 25 °C. The pellets containing microtubule-bound TPX2^α5−α7^ were retained after removal of the supernatant containing unbound TPX2^α5−α7^. The supernatant and a small amount of pellets resuspended in phosphate buffer were used for SDS-PAGE analysis.

TPX2^α5−α7^/microtubule assemblies were fully packed into the 1.3 mm and 0.7 mm zirconia ceramic Bruker MAS rotors. 3.8 mg of hydrated protonated U-[^13^C,^15^N]-TPX2^α5−α7^/MT pellets and 3.7 mg of hydrated deuterated U-[^13^C,^15^N]-TPX2^α5−α7^/MT assemblies were packed into two 1.3 mm fast MAS rotors. The deuterated U-[^13^C,^15^N]-TPX2^α^^5^^-^^α^^7^/MT sample was also packed in a 0.7 mm ultrafast MAS rotor, and the amount of hydrated pellets in the 0.7 mm rotor was ca. 0.8 mg.

### Solution NMR spectroscopy

The 2D ^1^H-^15^N HMQC solution NMR spectra of U-[^13^C,^15^N]-TPX2^α5−α7^, TPX2^α5-α7Δ^, and TPX2^α5-α6Δ^ were acquired on a 18.8 T Bruker Avance spectrometer (^1^H Larmor frequency of 800.3 MHz). The 2D heteronuclear spectra were acquired with 64 scans for TPX2^α5−α7^ and 128 scans for TPX2^α5-α7Δ^ and TPX2^α5-α6Δ^ using the SOFAST-HMQC^67^ scheme. This scheme employs recycle delays as short as 0.1 s for fast repetition rates and sensitivity gain. 256 complex points were collected in t_2_ dimension (^15^N) and the acquisition time is 43 ms.

The 2D ^1^H-^15^N HSQC and TROSY solution NMR spectra of U-[^13^C,^15^N]-TPX2^α5−α7^ in monodisperse state and as a condensate in solution were acquired on a 14.1 T Bruker Avance III spectrometer (^1^H Larmor frequency of 600.1 MHz) equipped with a triple-resonance inverse (TXI) detection probe. For monodisperse TPX2^α5−α7^, the 2D ^1^H-^15^N and ^1^H-^13^C HSQC spectra were acquired with 32 and 64 transients, respectively. 360 complex points were collected in the ^15^N dimension and the acquisition time is 49 ms. The ^1^H-^15^N TROSY experiments were performed with same parameters as the HSQC. The 2D ^1^H-^15^N HSQC spectrum of TPX2^α5−α7^ condensates in solution was acquired with 128 transients. For all the ^1^H-^15^N experiments, ^13^C decoupling was applied using the GARP decoupling scheme during the acquisition in ^15^N. WATERGATE scheme was used for water suppressions. The recycle delay was 3 s. All spectra were acquired at 296 K. Chemical shifts were referenced to 4,4-dimethyl-4-silapentane-1-sulfonic acid (DSS).

### MAS NMR spectroscopy

Multidimensional MAS NMR experiments were performed to obtain site-specific structural information for TPX2^α5−α7^ assembled with microtubules. The dipolar-based ^1^H- and ^13^C-detected MAS NMR spectra of fully protonated U-[^13^C,^15^N]-TPX2^α5−α7^/MT assemblies were acquired on a 14.1 T instrument (^1^H Larmor frequency of 599.8 MHz) outfitted with a narrow-bore Magnex magnet, a Bruker Avance III HD console and a 1.3 mm HCN triple-resonance MAS probe. The 2D ^13^C-detected homonuclear and heteronuclear correlated spectra, including the combined R2_n_^v^-driven (CORD)^68^, NCACX, NCOCX, and radio-frequency-driven dipolar recoupling (RFDR)^69^ spectra, were acquired at MAS frequency of 14 kHz controlled to within ±10 Hz by a Bruker MAS III controller. The actual sample temperature was approximately 3 °C, maintained to within ±0.1 °C by a Bruker temperature controller. The temperature was calibrated using KBr as the temperature sensor^70^. Typical 90° pulse lengths were 1.8 μs for ^1^H, 2.0 μs for ^13^C, and 3.3 μs for ^15^N. The ^1^H-^13^C and ^1^H-^15^N cross-polarization (CP) employed a linear amplitude ramp of 80–100% on ^1^H; the center of the ramp  was matched to Hartmann-Hahn conditions at the first or second spinning sideband.

The 2D ^13^C-^13^C CORD spectrum with a mixing time of 50 ms was acquired with 272 transients and an acquisition time of 4.5 ms in t_2_ dimension. The ^1^H radio frequency (rf) fields during the CORD homonuclear mixing were set to the spinning frequency (ν_r_) and half of ν_r_. ^1^H-^13^C CP was performed at zero-quantum (ZQ) condition with the rf fields of 106 kHz on ^1^H and 77 kHz (5.5 ν_r_) on ^13^C; a typical CP contact time was 0.8 ms.

2D ^13^C-^13^C MAS NMR experiments with longer homonuclear mixing times were also performed to obtain long-range restraints that inform on the tertiary structure of TPX2^α5−α7^ bound to microtubules. The 2D CORD spectra of U-[^13^C,^15^N]-TPX2^α5−α7^/MT with a mixing time of 200 ms were acquired at estimated sample temperatures of 3 °C and 15 °C with 368 and 400 transients, respectively. Typical acquisition time was 4.8 ms in t_2_. A number of long-range inter-residue correlations in both aliphatic and aromatic regions were exclusively detected in the CORD spectra with 200 ms mixing (Fig. 6).

The 2D NCOCX and NCACX heteronuclear spectra were acquired with 50 ms ^13^C-^13^C mixing using dipolar-assisted rotational resonance (DARR) recoupling^71^. The ^1^H-^15^N ZQ-CP was conducted with an rf field of 82 kHz (5.5 times the spinning rate ν_r_) on ^1^H and 62 kHz on ^13^C; the CP contact times for ^1^H-^15^N and ^15^N-^1^H CP transfers were 1.5 and 1.9 ms, respectively. The ^15^N-^13^C SPECIFIC-CP^72^ transfer was performed  with a tangent gradient^73^ of 90–110% applied on ^15^N channel; the rf fields were 61 kHz on ^15^N and 57 kHz on ^13^C; the CP contact time was 6 ms. The ^13^C frequency was set to 55 ppm during SPECIFIC-CP for NCACX and 170 ppm for NCOCX. The NCOCX spectrum was acquired with 2k transients and the acquisition time was 5.1 ms in t_2_. The NCA spectra were acquired with 704 transients and an acquisition time of 6.0 ms in ^15^N dimension. For all the ^13^C-detected experiments, SPINAL64 decoupling scheme^74^ was applied at a ^1^H rf field of 147 kHz during the t_1_ acquisition and SPECIFIC-CP. High-power decoupling ensures efficient removal of strong ^1^H-^1^H couplings.

The 2D ^1^H-detected CH HETCOR spectra of fully protonated TPX2^α5−α7^/MTs were acquired at a MAS frequency of 60 kHz with 64 transients. The sample temperature was approximately 15 °C. ^1^H-^13^C DQ-CP was performed with rf fields of 125 kHz on ^1^H and 85 kHz on ^13^C; the contact time was typically 0.8 ms for ^1^H-^13^C CP and 0.4–0.85 ms for ^13^C-^1^H CP transfers.

### ^1^H-detected MAS NMR experiments on deuterated U-[^13^C,^15^N]-TPX2^α5-α7^/MT

High-resolution 2D ^1^H-detected MAS NMR spectra of deuterated U-[^13^C,^15^N]-TPX2^α5-α7^/MTs assemblies were acquired on a 20.0 T Bruker Avance III spectrometer (^1^H Larmor frequencies of 850.4 MHz) outfitted with a 0.7 mm HCND MAS probe. The 2D heteronuclear correlated (HETCOR) spectra were acquired at ultrafast MAS frequencies of 100 kHz and 110 kHz controlled within ±20 Hz by a Bruker MAS III unit. The apparent temperatures were 243 K and 238 K maintained at ±0.1 °C by a BCU-III unit for 100 kHz and 110 kHz MAS, respectively. Typical 90° pulse lengths were 1.1 μs for ^1^H, 3.7 μs for ^13^C, and 2.7 μs for ^15^N. The ^1^H-^13^C CP transfer was performed at double-quantum (DQ) CP condition with a linear ramp of 90–110% applied on ^1^H and spin-lock rf fields at 2/3 ν_r_ for ^13^C and 4/3 ν_r_ for ^1^H; the Hartmann-Hahn condition was matched at two times the MAS frequency. The rf field strength was 162 kHz for ^1^H and 74 kHz for ^13^C at the MAS frequency of 110 kHz. Typical contact time for ^1^H-^13^C CP transfer is 2.1 ms. MISSISIPPI scheme was applied for water suppressions at ^1^H rf field of 10 kHz with a delay of 0.1 s and XY4 phase cycling. The recycle delay is 2 s. The experimental time for 2D CH HETCOR is typically 18h.

To determine which fast MAS regime is most efficient for the 3D ^1^H-detected experiments on deuterated TPX2^α5−α7^/MTs, we compared the sensitivities of 2D hCH HETCOR spectra acquired at MAS frequencies of 110 kHz and 60 kHz using the 0.7 mm and 1.3 mm MAS probes, respectively (Supplementary Fig. 5a, b). The sensitivity per milligram of sample at ultrafast MAS of 110 kHz is more than 2 times higher than that at 60 kHz MAS. Despite of that, the absolute signal-to-noise ratios of the former are lower than that of the latter (Supplementary Table 2). Therefore, we chose the experimental regime at 60 kHz MAS as it benefits from larger sample amount without significant deterioration in resolution for the deuterated sample.

The 2D and 3D ^1^H-detected spectra of deuterated U-[^13^C,^15^N]-TPX2^α5-α7^/MT, including hCANH, hCONH, and h(CO)CA(CO)NH^75^, were acquired on a 20.0 T Bruker Avance III spectrometer equipped with a 1.3 mm Bruker HCN MAS probe. The MAS frequency was 60 kHz maintained at ±10 Hz by a Bruker MAS III controller. Typical 90° pulses were 1.7 μs for ^1^H, 2.2 μs for ^13^C, and 2.3 μs for ^15^N. The experiments were performed at 243 K maintained at ±0.1 °C and the sample temperature is approximately 10 °C. The ^1^H-^13^C and ^1^H-^15^N ZQ-CP transfers were performed with a linear ramp of 90–110% on ^1^H; the center of ramp was matched to Hartmann-Hahn conditions at first spinning sideband. Typical rf field strengths were 41 kHz (2/3 ν_r_) for ^1^H and 116 kHz for ^13^C. The 2D hNH HETCOR spectrum was acquired with 96 scans with optimized contact times of 0.8 ms and 0.6 ms for the H-N and N-H CP transfers, respectively. Two 2D hCH HETCOR spectra were acquired at 245 K with a sample temperature of 12 °C; the ^1^H-^13^C CP contact times were 2.1/2.1 ms and 2.8/2.7 ms, respectively.

For the 3D ^1^H-dectected MAS NMR spectra, ^1^H-^15^N and ^1^H-^13^C CP were conducted with rf fields at 47 kHz (3/5 ν_r_) on ^15^N or ^13^C and 104 kHz (3/5 ν_r_ + 1 ν_r_) on ^1^H. The CP contact times for ^1^H-^13^C and ^15^N-^1^H transfers were 3.0–3.8 ms and 1.0–2.0 ms, respectively. The ^13^C-^15^N SPECIFIC-CP was conducted with ^13^C rf field at 45 kHz and ^15^N rf field at 37 kHz with a tangent ramp on ^15^N; typical contact times were 10–14 ms. The 3D hCANH and hCONH spectra were acquired with 64 transients and the h(CO)CA(CO)NH spectrum was acquired with 512 transients. Gaussian Q3.2000 frequency-selective pulse^76^ was used for the CO-CA and CA-CO selective transfers in the h(CO)CA(CO)NH experiment. For all the ^1^H-detected experiments, SW_f_-TPPM decoupling scheme^77^ at ^1^H rf field of 13 kHz was used for ^1^H decoupling during the acquisition in indirect dimensions. WALTZ-16^78^ decouplings were applied for ^13^C and ^15^N at a rf field of 25 kHz during ^1^H acquisition. ^1^H decoupling was not applied during the ^13^C-^15^N DCP. The MISSISIPPI water suppression was performed with a ^1^H rf field of 15 kHz and a delay of 0.18–0.2 s. The recycle delay was 1.7–2.0 s for 2D HETCOR and 2.0 s for the 3D spectra. The total experimental time was 3.5 days for hCANH, 4.4 days for hCONH, and 3.8 days for h(CO)CA(CO)NH. The 2D ^13^C-^13^C RFDR spectrum with direct ^13^C polarization was acquired with a RFDR mixing of 2.4 ms and 608 transients.

Detailed conditions for the ^13^C- and ^1^H-detected experiments are listed in Supplementary Table 3. The parameters for the ^1^H-detected experiments on deuterated TPX2^α5-α7^/MTs are summarized in Supplementary Table 4. The pulse sequences and optimization protocol of the experimental parameters for 3D ^1^H-detected experiments were tested and validated using U-[^13^C,^15^N]-MLF tripeptide as a model compound.

#### REDOR-filtered MAS NMR experiments

The ^13^C-detected double-REDOR filter-based MAS NMR experiments^58^ were performed on deuterated U-[^13^C,^15^N]-TPX2^α5−α7^ assembled with natural abundant microtubules at 14.1 T. All the dREDOR-based spectra were acquired at a MAS frequency of 14 kHz and a sample temperature of 5 °C. The 1D ^1^H-^13^C dREDOR-CPMAS spectra were collected with simultaneous ^1^H-^13^C and ^1^H-^15^N REDOR filters with dephasing times ranging from 0.14 ms (2τ_r_) to 2.0 ms (28τ_r_). For each 1D experiment, the S_0_ control spectrum was collected with same duration of zero quantum relaxation without REDOR dephasing. The S and S_0_ spectra were acquired with same transients: typically, 8 k for dephasing times of 0.43–1.14 ms and 10 k for dephasing times of 1.43–2.0 ms. For enhanced sensitivity, the 1D CPMAS spectra with dREDOR dephasing of 0.43 ms, 0.57 ms, and 0.86 ms were also acquired with 36 k, 34 k, and 36 k transients, respectively. The 2D ^13^C-detetcted dREDOR-HETCOR spectrum was acquired with a dephasing time of 0.43 ms with 6912 transients; 2048 and 42 complex points were collected in t_1_ and t_2_, respectively. The recycle delay was 2 s. The 1D and 2D CP-based experiments without dipolar filters were also performed for comparison. The 1D direct ^13^C polarization and ^1^H-^13^C CPMAS spectra were acquired with 2 k and 8 k transients, respectively. The recycle delay was 6 s for the direct polarization experiment. The 2D ^1^H-^13^C HETCOR spectrum was acquired with 1280 transients. The ^1^H-^13^C CP contact time was 6 ms for the 2D HETCOR and dREDOR-filtered experiments.

The proton magnetizations from TPX2^α5−α7^ molecules are dephased by dREDOR dipolar filters, and intermolecular correlations are established via dipolar-based transfers from ^1^H in microtubules to proximal ^13^C atoms in TPX2^α5−α7^. As the TPX2^α5−α7^ protein is uniformly ^13^C- and ^15^N-enriched with a high level of deuteration and proton back-substitutions at exchangeable amide sites, the duration of dREDOR dephasing required for a complete removal of protons in TPX2^α5−α7^ is significantly shorter than that for fully protonated proteins as was previously reported^58,59^. All the Cα signals and majority of Cβ signals of TPX2^α5−α7^ are dephased with a dREDOR dephasing time of 0.43 ms, which leads to their absence in the 1D dipolar filtered MAS NMR spectra (Fig. 5a, Supplementary Fig. 12). These results indicate that the amide ^1^H signals of TPX2^α5−α7^ in the condensates on MTs were fully dephased under the chosen conditions. Furthermore, the same set of peaks was retained in the 1D dREDOR-CPMAS spectra with various dephasing times of up to 1.43 ms, indicating that these ^13^C signals arise from long-range correlations (Supplementary Fig. 12). There are a few remaining signals involving the dynamic Lys sidechains and the intense signals at ^13^C chemical shifts of 66 and 76 ppm from the solvent, which were not identified as intermolecular contacts with MTs. The ^1^H resonances arising from MTs were tentatively assigned based on the chemical shifts of α and β tubulin predicted by SHIFTX2^63^ (Supplementary Table 8).

### NMR data processing and spectral analysis

All the ^13^C-detected spectra were processed in NMRPipe^79^ and analyzed using CcpNmr analysis^80^. The 2D CORD and RFDR spectra were processed with 40/80 Hz line-broadening Gaussian apodization in both dimensions for sensitivity enhancement or with 30-, 60-, or 90-degree shifted sinebell apodization for resolution enhancement. The ^1^H-detected NMR spectra were processed in Topspin with 30-, 60-, and 90-degree shifted sine-bell apodization. The 2D hCH HETCOR acquired at ultrafast MAS frequencies of 100–110 kHz were processed without apodization.

For atom-specific resonance assignments, we used the 2D ^13^C-detected and 3D ^1^H-detected spectra acquired on two TPX2^α5−α7^/MT assemblies prepared with fully protonated or deuterated TPX2^α5−α7^ (Supplementary Fig. 3). These included 2D CORD (Supplementary Fig. 2b), NCA, NCACX, and NCOCX spectra (Supplementary Fig. 4), in combination with 3D hCANH and h(CO)CA(CO)NH spectra (Fig. 3c, d, Supplementary Fig. 5e). Backbone walk was carried out using the 3D spectra. The MAS NMR assignments were facilitated by the HMQC solution NMR spectra of the TPX2^α5-α7^, TPX2^α5-α7^^Δ^ and TPX2^α5-α6Δ^ constructs, which resolved many ambiguities and enabled unambiguous identification of the residues removed by truncations. One such example is Gly residues (a total of four in the TPX2^α5−α7^), as illustrated in Fig. 2b, c. By comparing the spectra of the three TPX2 constructs, resonances belonging to G489 and G570 were uniquely assigned and the peaks associated with G622 and G628 were subsequently assigned. The correlations in the CORD spectra were partially assigned where unambiguous assignments of Cα shifts are available. The inter-residue correlations between aromatic and aliphatic sidechains helped to reduce assignment ambiguities.

We assigned the well-resolved amide-sidechain correlations in the 2D CH HETCOR spectra of deuterated TPX2^α5−α7^/MTs acquired with longer CP contact time. A number of medium- and long-range inter-residue ^13^C-^1^H contacts were identified (Supplementary Fig. 11a, b) and ambiguities were resolved using the 3D structure. These restraints correspond to typical interatomic distances of 3–8 Å in the MAS NMR structure of TPX2^α5−α7^ bound to MTs (Supplementary Fig. 11c) and validate the sidechain conformations of corresponding residues in multiple functional motifs and loop regions in TPX2^α5−α7^.

### Modeling and structure calculation of TPX2^α5−α7^ bound to MTs

The initial models of TPX2^α5−α7^ were generated from homology-based structure prediction by Robetta^43^. The primary amino acid sequence was provided and five structural models were predicted under the Rosetta Ab Initio mode^81,82^. The predicted structural models were evaluated using the medium- and long-range experimental restraints. Models that have general consistency with the experimental results were used for the structure calculations and the ones that significantly contradict with the experimental restraints were ruled out. Structural model of TPX2^α5−α7^ was also predicted by AlphaFold 2 as a comparison.

The NMR structure calculations were performed in Xplor-NIH (version 2.53)^68,83,84^ using the TPX2^α5−α7^ model generated from Robetta prediction as the starting model and the ^13^C-^13^C distance restraints and backbone dihedral angle restraints derived from MAS NMR experiments. In total, there were 341 unambiguous and 1 ambiguous ^13^C-^13^C distance restraints. Unambiguous and ambiguous internuclear distance restraints were obtained from the assigned correlations in the 2D ^13^C-^13^C CORD spectra. Ambiguous restraints exceeding 5-fold ambiguity were not considered. Bounds of the distance restraints were set to 1.5–6.5 Å (4.0 ± 2.5 Å) and 2.0–7.2 Å (4.6 ± 2.6 Å) for intra- and inter-residue restraints, respectively, consistent with our previous studies^85–87^. Dihedral restraints were predicted using TALOS-N^88^ from the experimental solid-state ^1^H, ^13^C and ^15^N chemical shifts.

Due to the sparse amount of distance restraints, we performed structure calculations with an implicit-solvent potential carried out with the EEFx module^89^ in Xplor-NIH to aid in the accuracy and precision. Calculations were initiated from the coordinates of the homology model described in the previous section and 100 structures were annealed. Residues with sparse or no distance restraints were set as rigid-bodies (513–526, 551–553, 562–583, 604–607, 611–617, 629–661, 670–680, 685–690, 692–694, 697–699, 479–484, 502–508, 591–602, 621–627, 681–684). Standard terms for bond lengths, bond angles, and improper angles were used to enforce correct covalent geometry. A statistical torsion angle potential^90^ was employed.

Following the recommended parameters in the EEFx example script, annealing was performed in the run at 3500 K for 15 ps or 15,001 steps, whichever completed first. The starting time step was 1 fs and was self-adjusted in subsequent steps to ensure conservation of energy. The initial velocities were randomized about a Maxwell distribution using the starting temperature of 3500 K. Subsequently the temperatures were reduced to 25 K in steps of 12.5 K. At each temperature, the initial time step was set to the default value of 1 fs, and a 0.4-ps dynamics run was performed. Force constants for distance restraints were ramped from 2 to 30 kcal mol^−1^ Å^−2^. The dihedral restraint force constants were set to 10 kcal mol^−1^ rad^−2^ for high-temperature dynamics at 3000 K and 200 kcal mol^−1^ rad^−2^ during cooling. The calculation was repeated two more times for a total of three iterations. The second and third iteration were initiated from the coordinates of the 10 lowest energy structures in the proceeding iteration. The above calculations were repeated multiple times to ensure reproducibility.

Root-mean-square (R.M.S.) deviation values were calculated using routines in Xplor-NIH. Restraint tallying and format conversions were carried out with in-house Python 2.7 scripts. The visualization of structural ensembles was rendered in PyMOL 1.8.4 using in-house shell/bash scripts. Secondary structure elements were classified according to TALOS-N and manual inspection.

Additional structure calculations were performed using modified protocols for rigid bodies, and similar structural models were generated. The structure calculated using a protocol that releases the rigid bodies during the energy minimization is generally consistent with the one calculated without releasing the rigid bodies (Supplementary Fig. 9).

### Molecular docking

The molecular models for TPX2^α5−α7^ bound to microtubule filaments were predicted by docking in ClusPro 2.0^91^ without any data-derived restraints. The Cryo-EM structure of microtubules (PDB 3J6G) and the MAS NMR structure of TPX2^α5−α7^ calculated in Xplor-NIH were used for the blind docking. The 10 top-ranked models based on the balanced mode were evaluated and adopted.

The molecular docking of TPX2^α5−α7^ on the βα tubulin heterodimer was performed in HADDOCK 2.4^92,93^ using the restraints derived from dREDOR-based experiments as ambiguous interaction restraints. We performed the docking using two types of tubulin dimers: one is alpha-beta tubulin heterodimer, and the other is beta-alpha dimer comprising alpha tubulin and the beta tubulin from the preceding heterodimer. Both structures were extracted from the cryo-EM structure of microtubules stabilized by taxol. Most TPX2 residues identified in the dREDOR MAS NMR spectra were included as the active sites for the docking in the ambiguous mode. The Lys residues and those that correlate with the ^1^H resonances of water were excluded. Several residues in the C-terminal regions of α-tubulin (D396, K401, S419, E423) and β-tubulin (T396, R401, T419, S423) were inputted as the active sites in MTs, which prevent incorrect docking of TPX2^α5−α7^ molecules at the inner side of microtubules. Both the blind docking in ClusPro and the data-guided docking in HADDOCK using MAS NMR restraints were repeated three times for reproducible results. The repeated dockings yield the same predicted binding modes of TPX2^α5−α7^ with the MT.

### Reporting summary

Further information on research design is available in the Nature Portfolio Reporting Summary linked to this article.

## Supplementary information

Supplementary information

Reporting Summary

## Data Availability

The MAS NMR structure of TPX2^α5−α7^ bound to microtubules and the coordinates have been deposited in the Protein Data Bank under accession code 8CX6. The MAS NMR chemical shifts are deposited in the Biological Magnetic Resonance Bank (BMRB) under BMRB entry ID 31025. The cryo-EM structure of paclitaxel-stabilized microtubules used in this study is available under PDB entry 3J6G.
