# Supplementary material for: Structural basis of protein condensation on microtubules underlying branching microtubule nucleation
Source: Nat Commun. 2023 Jun 21;14:3682. doi: 10.1038/s41467-023-39176-z (PMC10284871; doi:10.1038/s41467-023-39176-z)
Supplement: Supplementary file 1 — Supplementary information [file 41467_2023_39176_MOESM1_ESM.pdf]

Supplementary Information for

Structural Basis of Protein Condensation on Microtubules Underlying Branching Microtubule Nucleation

Changmiao Guo[1#], Raymundo Alfaro-Aco[2#], Chunting Zhang[1], Ryan W. Russell[1], Sabine Petry[2,*], Tatyana Polenova[1,*]

[1]*Department of Chemistry and Biochemistry, University of Delaware, Newark, Delaware 19716, United States*
[2]*Department of Molecular Biology, Princeton University, Princeton, New Jersey 08544, United States*

[#] *These authors contributed equally to this work.*

***Corresponding authors:** Sabine Petry, Email: spetry@princeton.edu; Tatyana Polenova, Email: tpolenov@udel.edu

Supplementary Figures

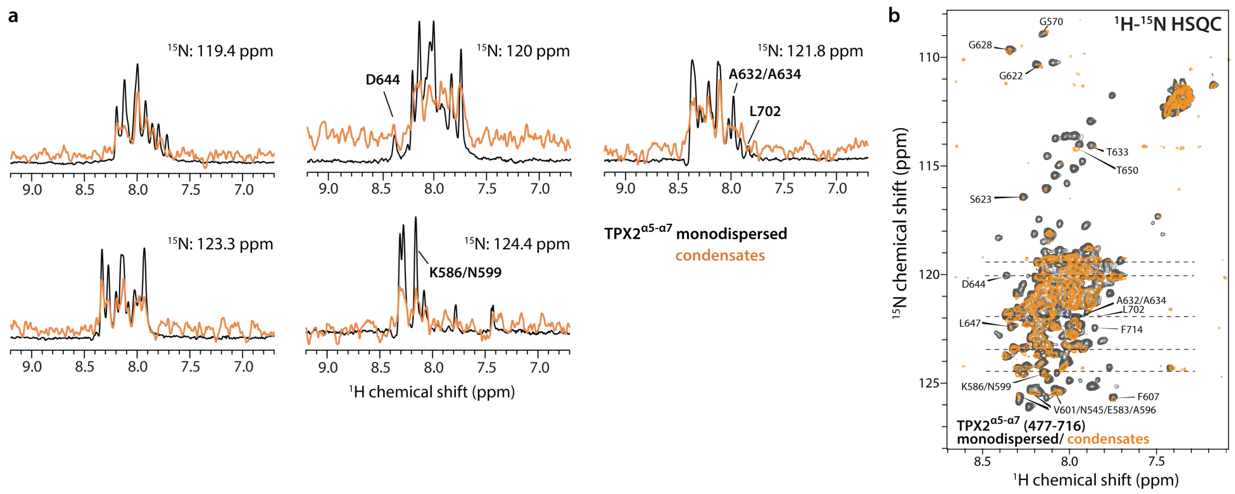

Supplementary Figure 1 | Line broadenings for TPX2$^{\alpha5\text{-}\alpha7}$ condensates in solution compared to the monodispersed solution. a Representative 1D ^{1}H traces and **b** 2D ^{1}H-^{15}N HSQC solution NMR spectra of free U-[^{13}C,^{15}N]-TPX2$^{\alpha5\text{-}\alpha7}$ in monodispersed solution (black) and condensates (orange). ^{1}H and ^{15}N line broadenings are observed for most detected signals of TPX2$^{\alpha5\text{-}\alpha7}$ condensates in solution. The 1D traces were extracted from the 2D HSQC spectra at ^{15}N shifts indicated by dashed lines in **b**.

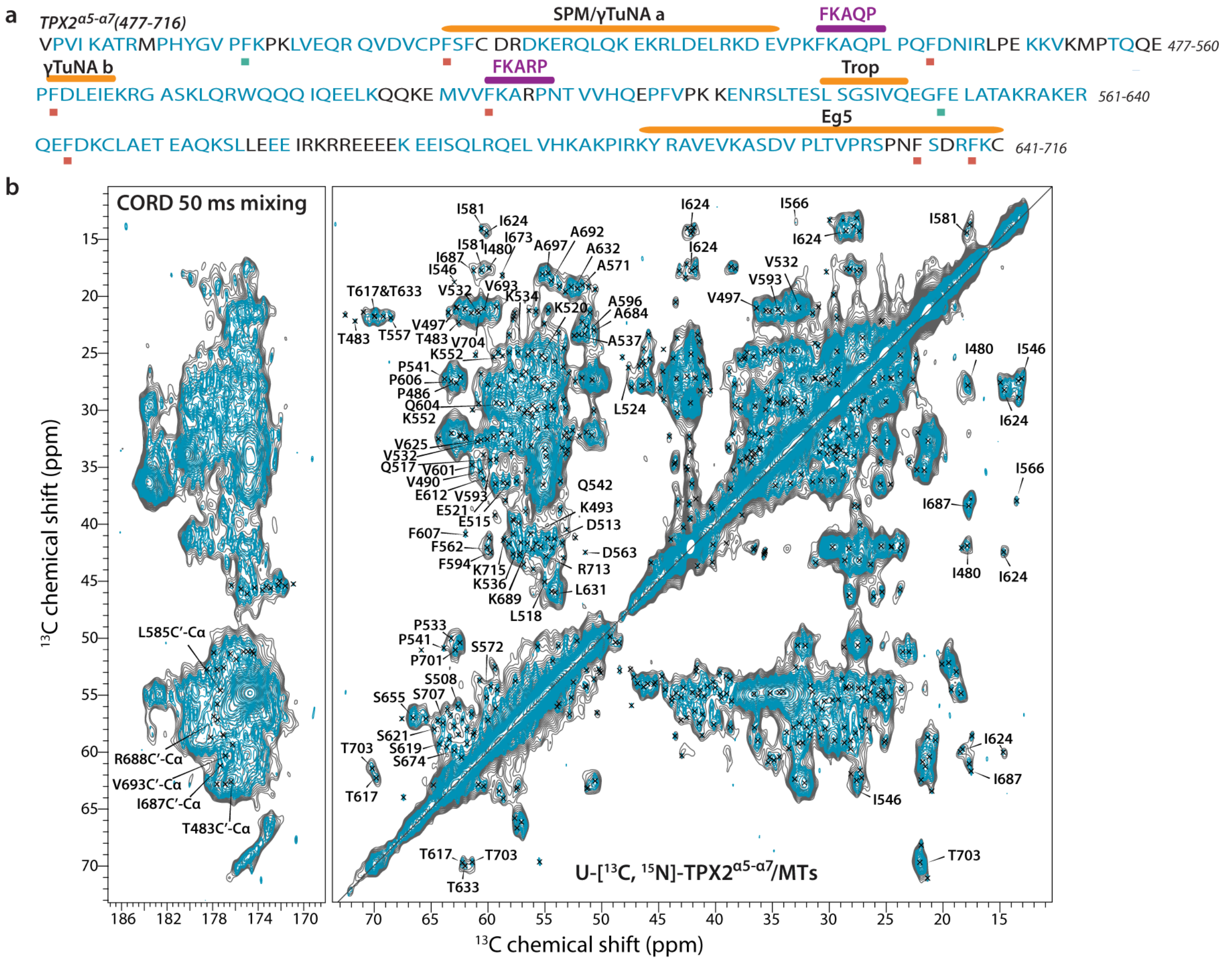

Supplementary Figure 2 | MAS NMR spectra of U-[^{13}C,^{15}N]-TPX2$^{\alpha5\text{-}\alpha7}$/MT assemblies and site-specific resonance assignments. a Mapping of assigned residues (cyan) onto the primary sequence of TPX2$^{\alpha5\text{-}\alpha7}$ (residues 477-716). The functional regions and regions of interests are indicated. The Phe residues where the single-site mutations severely affect TPX2 activity in stimulating branching microtubule nucleation and those that have no effect are marked by red and green dots, respectively. **b** 2D ^{13}C-^{13}C homonuclear correlated spectra of fully protonated U-[^{13}C,^{15}N]-TPX2$^{\alpha5\text{-}\alpha7}$/MT assemblies, acquired with 50 ms CORD mixing. Representative resonance assignments are labeled. The spectrum was processed with 90- (gray) and 60-degree (cyan) shifted sine-bell apodization. The spectrum was acquired at a magnetic field of 14.1 T using the sample of 3.8 mg TPX2$^{\alpha5\text{-}\alpha7}$/MT assemblies in an 1.3 mm MAS rotor; the MAS frequency was 14 kHz.

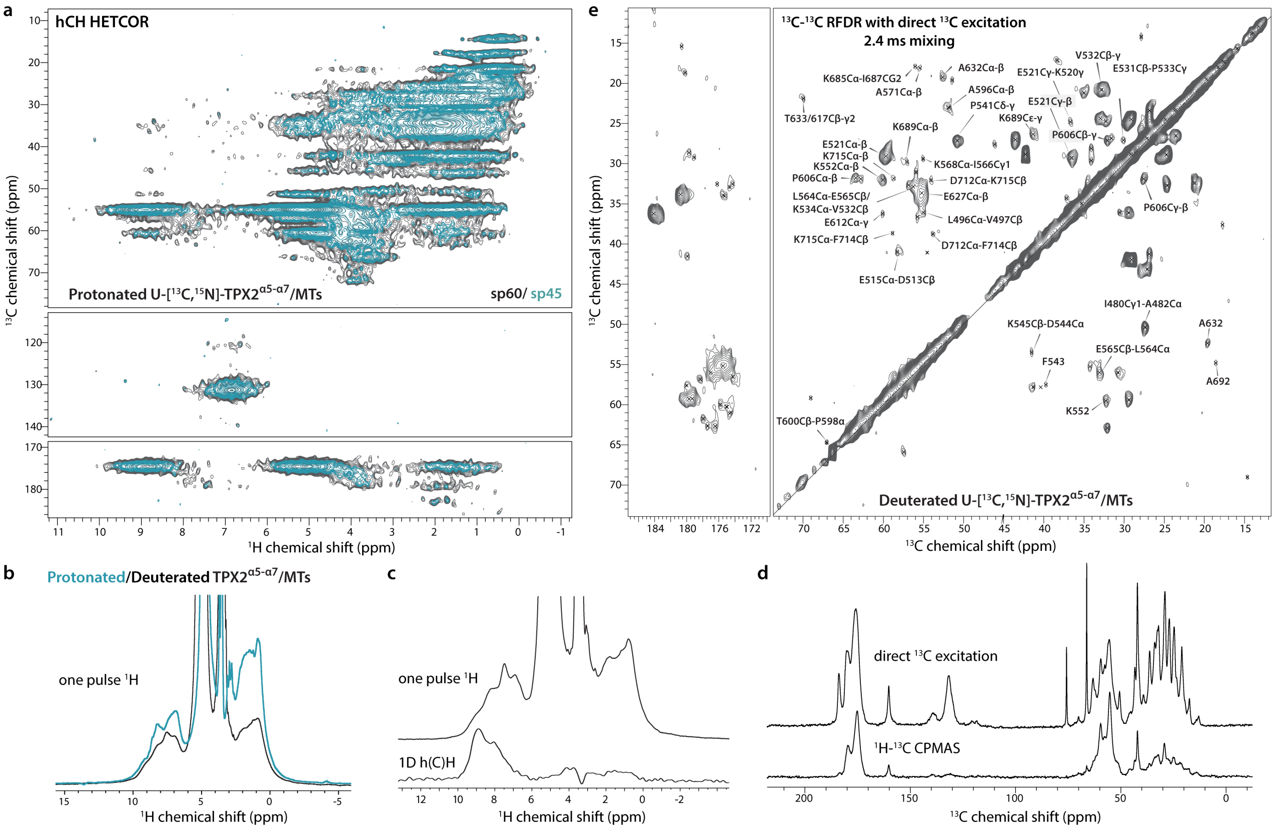

Supplementary Figure 3 | ^{1}H and ^{13}C-detected MAS NMR spectra of protonated and deuterated TPX2$^{\alpha5\text{-}\alpha7}$ in assemblies with MTs. a 2D ^{1}H-detected CH HETCOR spectra of protonated U-[^{13}C,^{15}N]-TPX2$^{\alpha5\text{-}\alpha7}$/MTs. The spectra were processed with 60º (gray) and 45º (teal) sine-bell shifted apodization for resolution enhancement. The contact times for ^{1}H-^{13}C and ^{13}C-^{1}H CP magnetization transfers were 0.8 ms and 0.85 ms, respectively. **b** Comparison of the 1D ^{1}H spectra of protonated (cyan) and deuterated (black) TPX2$^{\alpha5\text{-}\alpha7}$/MTs. The exchangeable amide sites in deuterated TPX2$^{\alpha5\text{-}\alpha7}$ were 100% back-substituted with protons. For deuterated TPX2$^{\alpha5\text{-}\alpha7}$/MTs, the ^{1}H signals primarily arise from the fully protonated microtubules, the back-exchanged amide protons and residual sidechain protons in TPX2$^{\alpha5\text{-}\alpha7}$ protein. The intensities of amide and sidechain protons decrease by 35% and 55% for the deuterated sample compared to the protonated sample, after a correction of signal-noise ratios based on the $\mathbf{B_0}$ field dependence. The 1D ^{1}H spectra were acquired with 16 transients. **c** 1D ^{1}H one pulse and h(C)H CPMAS spectra of deuterated TPX2$^{\alpha5\text{-}\alpha7}$/MTs, acquired with 4 and 144 transients, respectively. **d**, **e** 1D and 2D ^{13}C-detected MAS NMR spectra of deuterated TPX2$^{\alpha5\text{-}\alpha7}$/MTs. The 1D ^{13}C direct polarization and ^{1}H-^{13}C CPMAS spectra (**d**) were collected with 2048 and 8096 transients, respectively. The 2D direct polarization ^{13}C-^{13}C spectrum (**e**) were acquired with 2.4 ms RFDR mixing. The spectra of deuterated and protonated TPX2$^{\alpha5\text{-}\alpha7}$/MTs were acquired at 20.0 T and 14.1 T, respectively. The 1D ^{1}H spectra of the deuterated sample were acquired at an ultrafast MAS frequency of 100 kHz using a 0.7 mm HCND MAS probe. All the other spectra were acquired at a fast MAS frequency of 60 kHz using a 1.3 mm HCN probe.

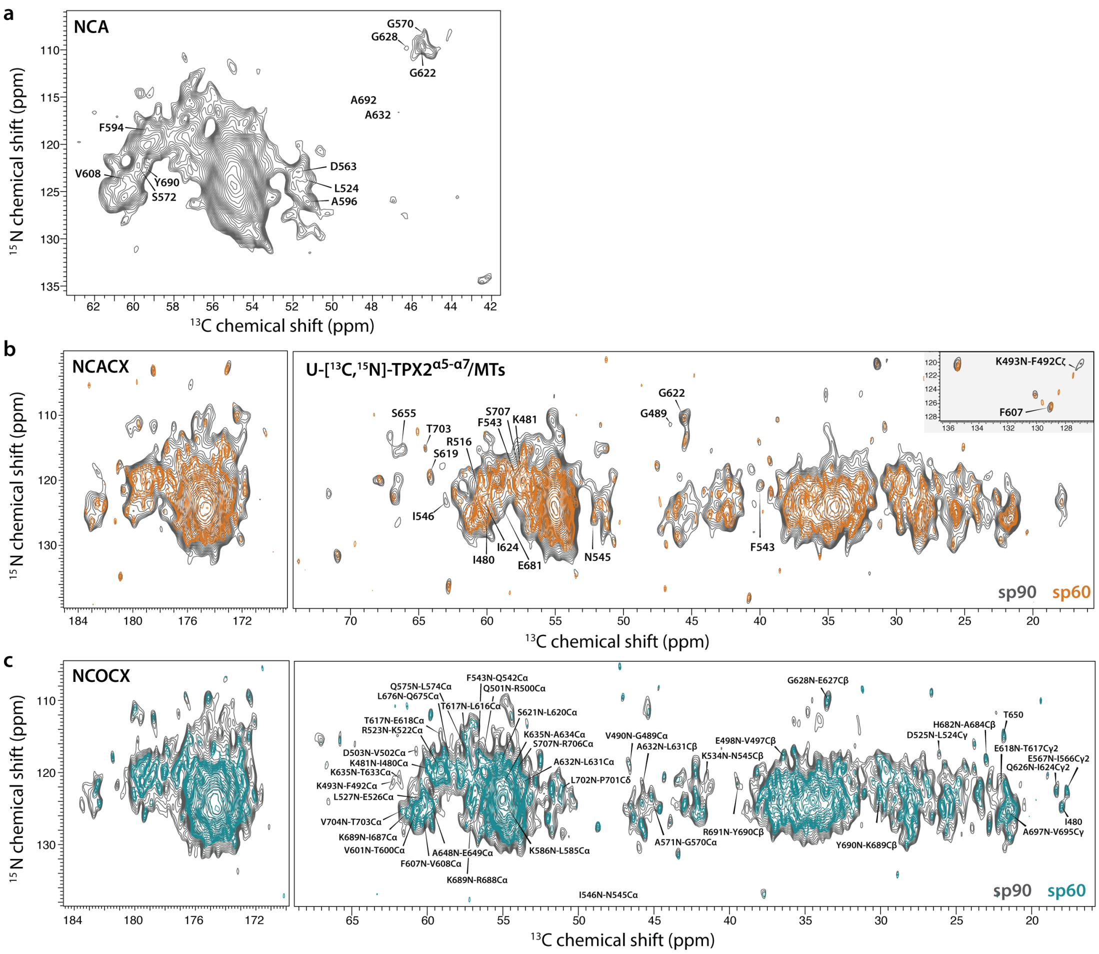

Supplementary Figure 4 | 2D ^{13}C-detected heteronuclear correlated MAS NMR spectra of fully protonated U-[^{13}C,^{15}N]-TPX2$^{\alpha5\text{-}\alpha7}$/MT assemblies. a 2D NCA spectrum, which reveals intra-residue correlations between backbone amide N and Cα atoms. **b** 2D NCACX and **c** NCOCX spectra, acquired with 50 ms ^{13}C-^{13}C mixing using the DARR recoupling scheme. The spectra reveal intra-residue correlations and sequential inter-residue correlations, respectively. Correlations of amide resonances with sidechain ^{13}C resonances were detected and identified. The spectra were processed with 90°- (gray) and 60°-degree (light orange and cyan) sine-bell shifted apodization.

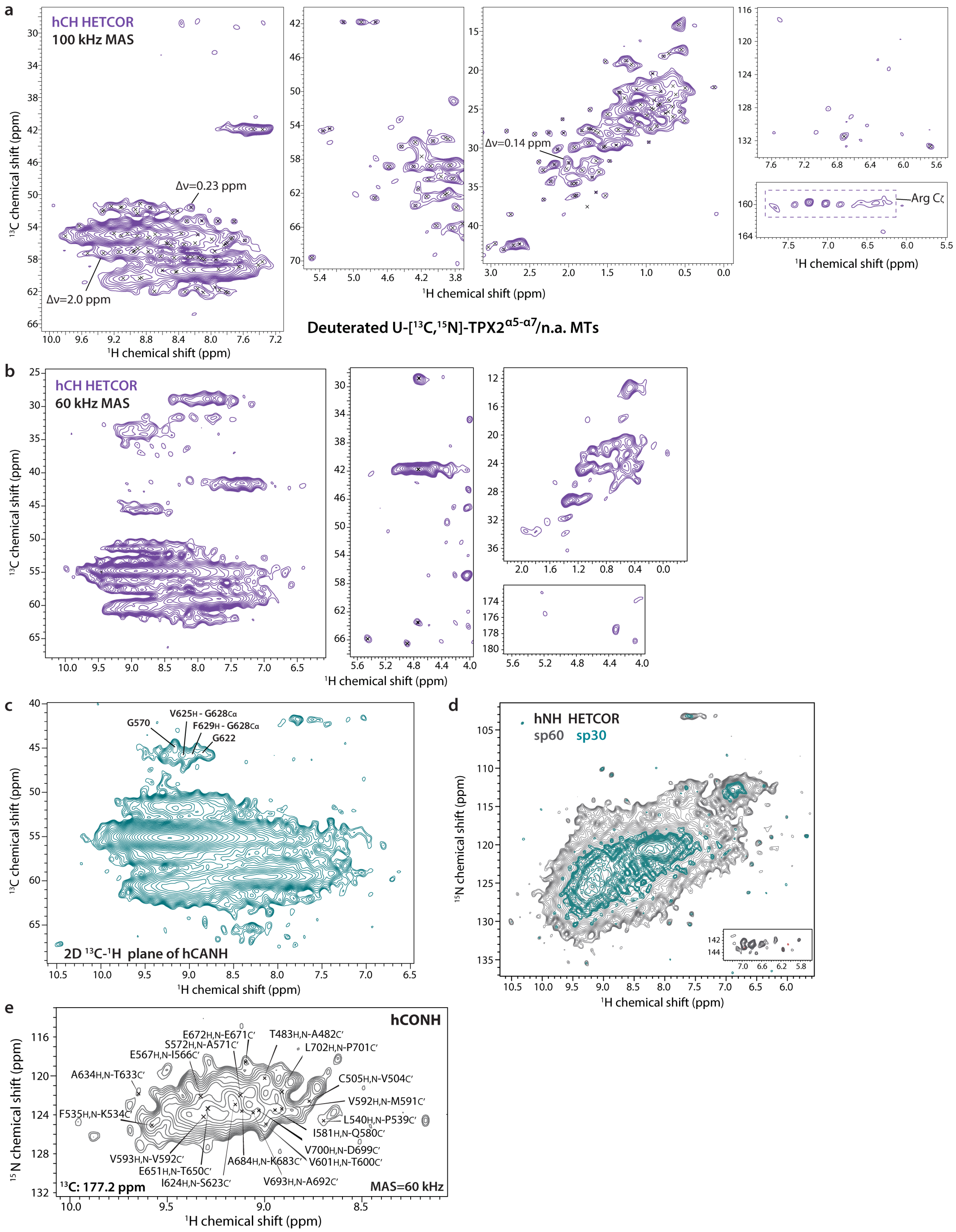

Supplementary Figure 5 | High-resolution ^{1}H-detected heteronuclear MAS NMR spectra of deuterated TPX2$^{\alpha5\text{-}\alpha7}$ in assemblies with MTs. a, **b** 2D ^{13}C-^{1}H HETCOR spectra acquired at ultrafast and fast MAS frequencies. Both spectra reveal signals of backbone amide protons and residual protonation in aliphatic sidechains. There is site-to-site variation in the ^{1}H line widths in both spectra, indicating the conformational heterogeneity of TPX2$^{\alpha5\text{-}\alpha7}$ condensates on MTs. The spectra were acquired at MAS rates of 100 kHz (**a**) and 60 kHz (**b**) using a 0.7 mm HCND probe and 1.3 mm HCN probe, respectively. The CP contact time for the ^{1}H-^{13}C and ^{13}C-^{1}H transfers was 2.1 ms. **c** 2D ^{13}C$_\alpha$-^{1}H correlated spectrum of the 3D hCANH experiment. The first 2D plane was acquired with 320 transients for higher signal-to-noise ratio and reveals a few signals that are absent in the 3D hCANH spectrum. In particular, several correlations of Gly residues were readily detected and identified. **d** 2D ^{15}N-^{1}H HETCOR spectra. The spectra were processed with 30°- (cyan) and 60°-degree (gray)

sine-bell shifted apodization. **e** Representative 2D ^{15}N-^{1}H plane of the 3D hCONH spectrum with sequential resonance assignments. All spectra were acquired at a magnetic field of 20.0 T; the MAS frequency was 60 kHz for spectra in **b-e**. The deuterated U-[^{13}C,^{15}N]-TPX2$^{\alpha5\text{-}\alpha7}$ was 100% ^{1}H back-exchanged and assembled with unlabeled MTs.

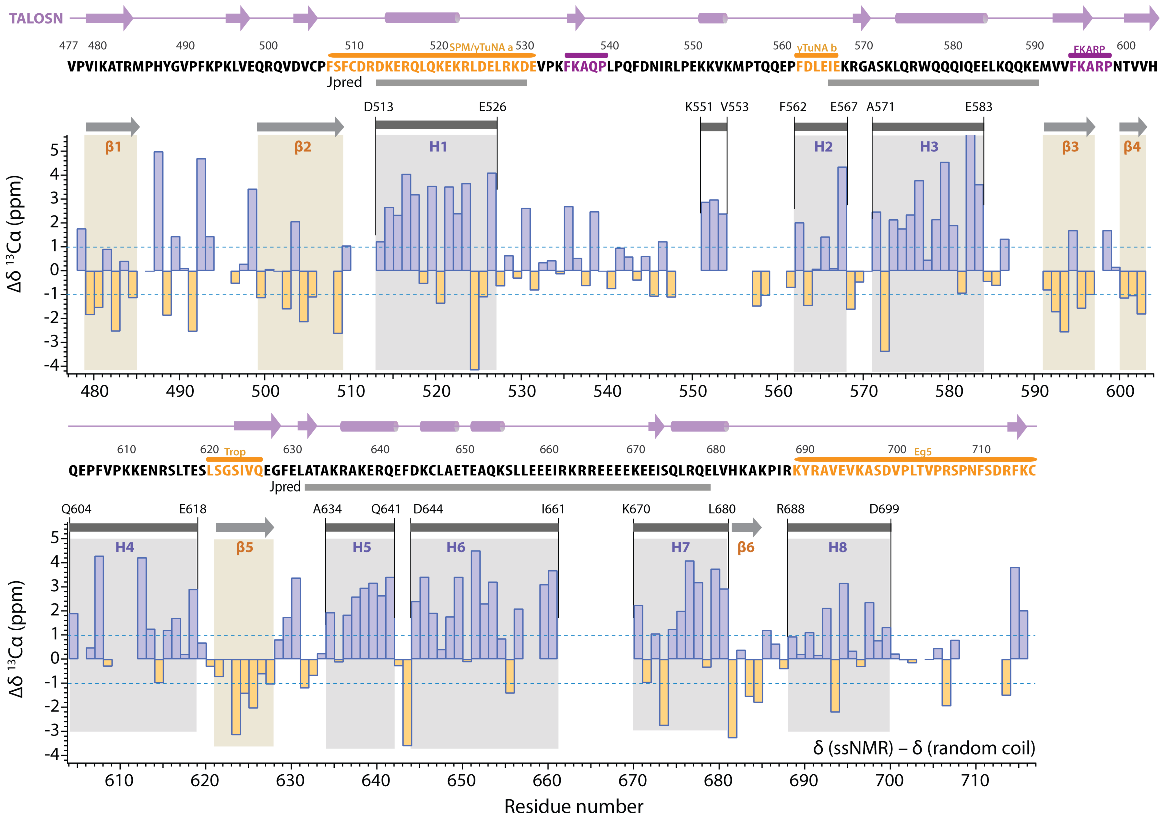

Supplementary Figure 6 | Chemical shift index for secondary structures of TPX2$^{\alpha5\text{-}\alpha7}$ bound to MTs. The secondary structural elements of TPX2$^{\alpha5\text{-}\alpha7}$ bound to MTs were derived from MAS NMR secondary chemical shifts of ^{13}Cα resonances. Positive and negative δΔ^{13}Cα values in a consecutive segment indicate the α-helix (purple) and β-sheet (yellow) structures, respectively. Regions with α-helical structures include the segments D513-E526, F562-E567, A571-E583, Q604-E618, A634-Q641, D644-I661, K670-L680 and R688-D699 (sequentially named helix 1-8, or H1-8). Residues at the edge of helices are defined with moderate confidence. Six segments with β-sheet structures (β1-6) were revealed and include two functional regions, the segment that contain FKARP motif and Trop region. The secondary structures derived from TALOS-N prediction are shown at the top (light purple). Cylinders and arrows represent helices and sheets, respectively. Thresholds at +1 ppm and -1 ppm are marked as dashed lines.

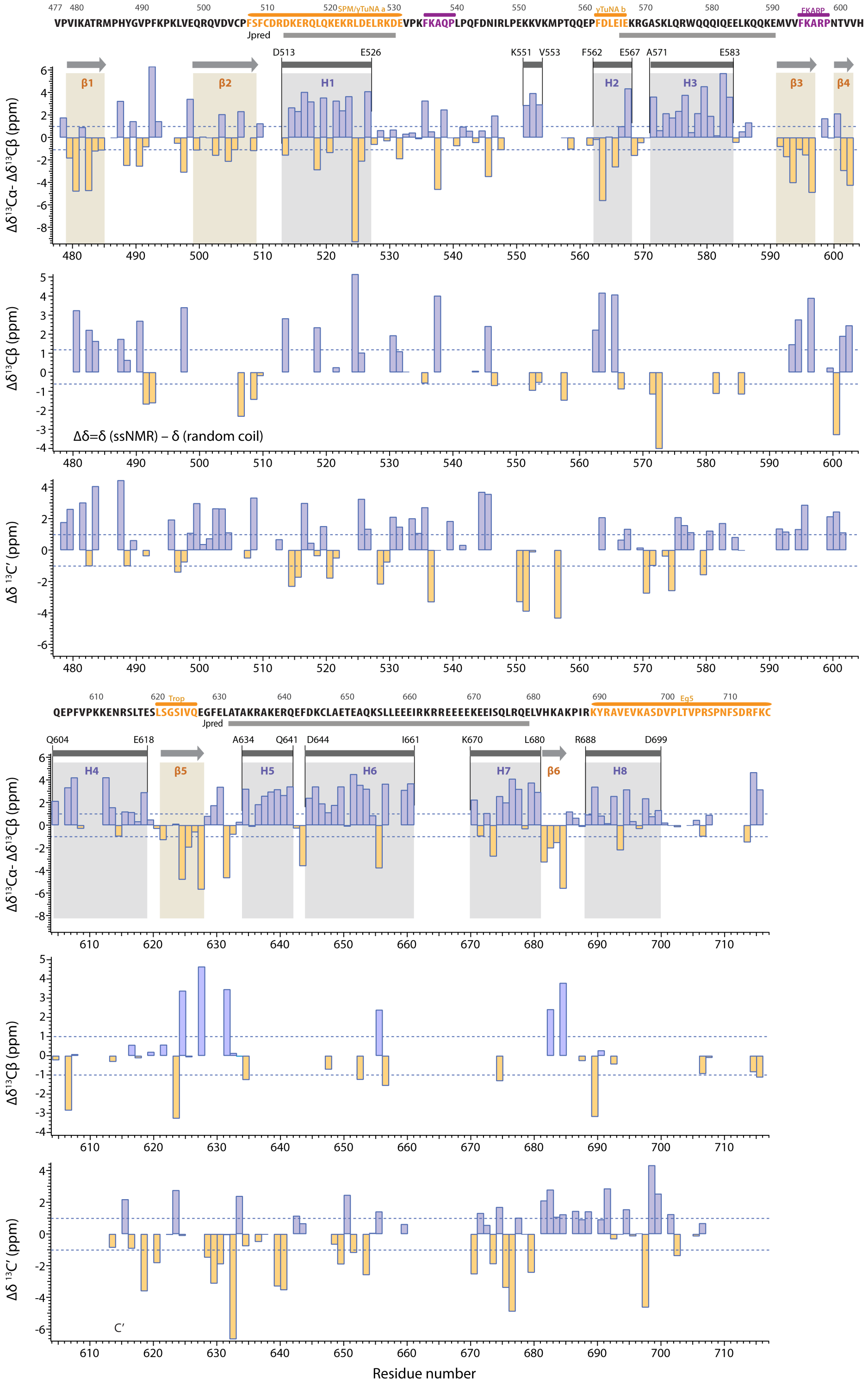

Supplementary Figure 7 | Chemical shift index of TPX2$^{\alpha5\text{-}\alpha7}$ bound to MTs. Secondary chemical shifts of $\Delta\delta^{13}C\alpha$-$\Delta\delta^{13}C\beta$, $\Delta\delta^{13}C\beta$ and $\Delta\delta^{13}C'$ were plotted vs. residue number. The secondary structures derived from $\delta\Delta^{13}C\alpha$-$\delta\Delta^{13}C\beta$ are consistent with those from $\delta\Delta^{13}C\alpha$. The only difference is that residues F562-E565 in γ-TuNA b motif show tendency to adopt β-sheet structure instead of α-helix.

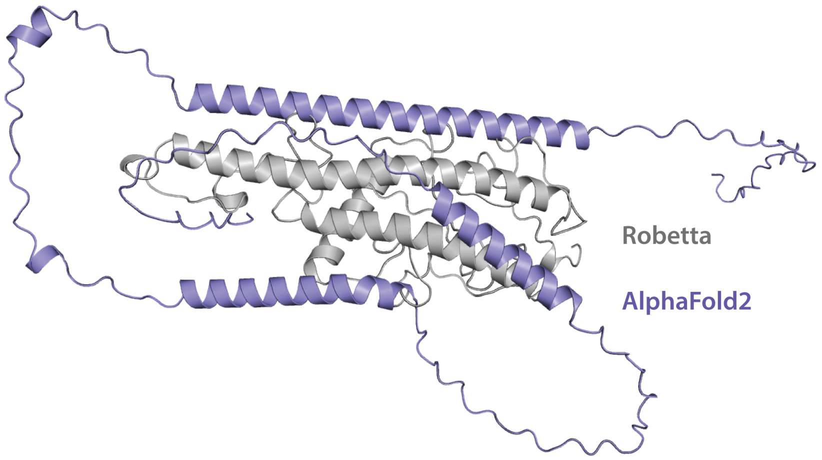

Supplementary Figure 8 | TPX2$^{\alpha5\text{-}\alpha7}$ models generated from Robetta (gray) and AlphaFold 2 (purple) predictions. Both homology-based structure predictions reveal three main helices that were previously known from other algorithms such as Jpred. The AlphaFold 2 prediction indicates an unfolded structure of TPX2$^{\alpha5\text{-}\alpha7}$, which is a clear discrepancy as compared to the MAS NMR experimental results.

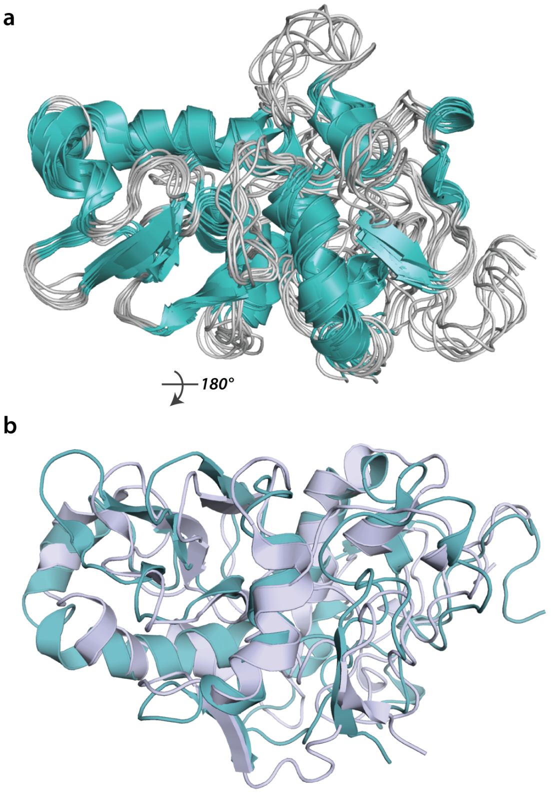

Supplementary Figure 9 | MAS NMR structure of TPX2$^{\alpha5\text{-}\alpha7}$ bound to MTs. a Ensemble of the 6 lowest energy structures. **b** Comparison of the medoids from structure calculations with (light purple) and without (teal) releasing the rigid bodies during the energy minimization.

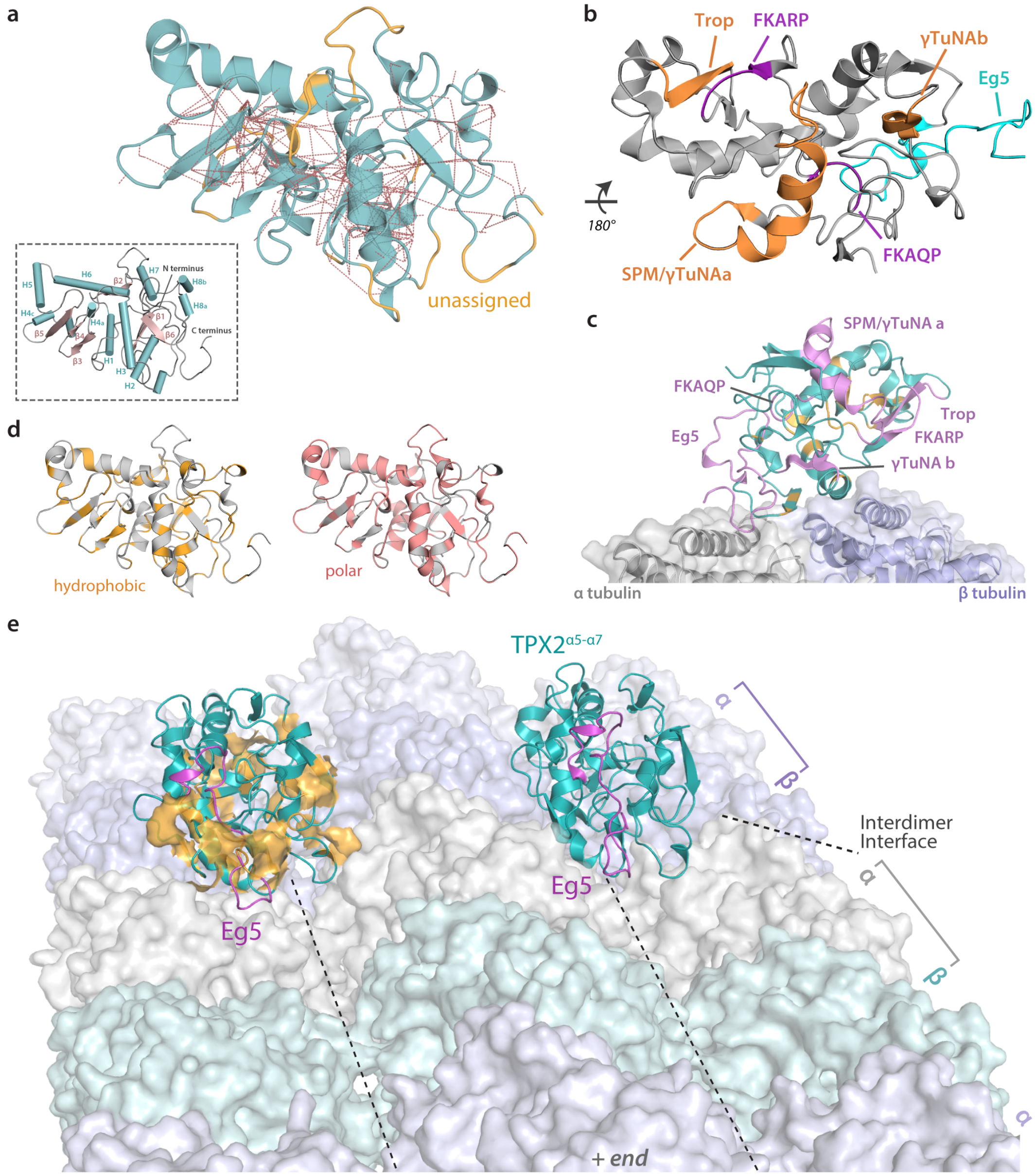

Supplementary Figure 10 | MAS NMR structure of TPX2$^{\alpha5\text{-}\alpha7}$ assembled on MTs. a Mapping of MAS NMR-derived intramolecular distance restraints (red dashed lines) on the structure of TPX2$^{\alpha5\text{-}\alpha7}$ bound to MTs. Unassigned residues are shown in light yellow. **b**, **c** Conformation of the functional regions and regions of interest in TPX2$^{\alpha5\text{-}\alpha7}$ bound to MT. TPX2$^{\alpha5\text{-}\alpha7}$ binds to MTs at the tubulin interdimer interface, with the γ-TuNA a and b motifs, the Trop region and FKARP motif positioned facing away from the MT. **d** Distributions of the hydrophobic (orange) and polar residues (salmon) in the 3D molecular structure of TPX2$^{\alpha5\text{-}\alpha7}$. The hydrophobic residues are evenly distributed, and a large portion of the polar residues are surface exposed. **e** Side view of MTs decorated with TPX2$^{\alpha5\text{-}\alpha7}$ (teal). The models are generated by molecular docking in ClusPro 2.0 without any experimental restraints. Majority of the residues determined by dREDOR-based experiments (orange surface) constitute the binding interface of TPX2$^{\alpha5\text{-}\alpha7}$ with MTs. The Eg5 region (magenta) remain exposed, which ensures its accessibility to Eg5. The α and β tubulin in tandem heterodimers are differentially color coded.

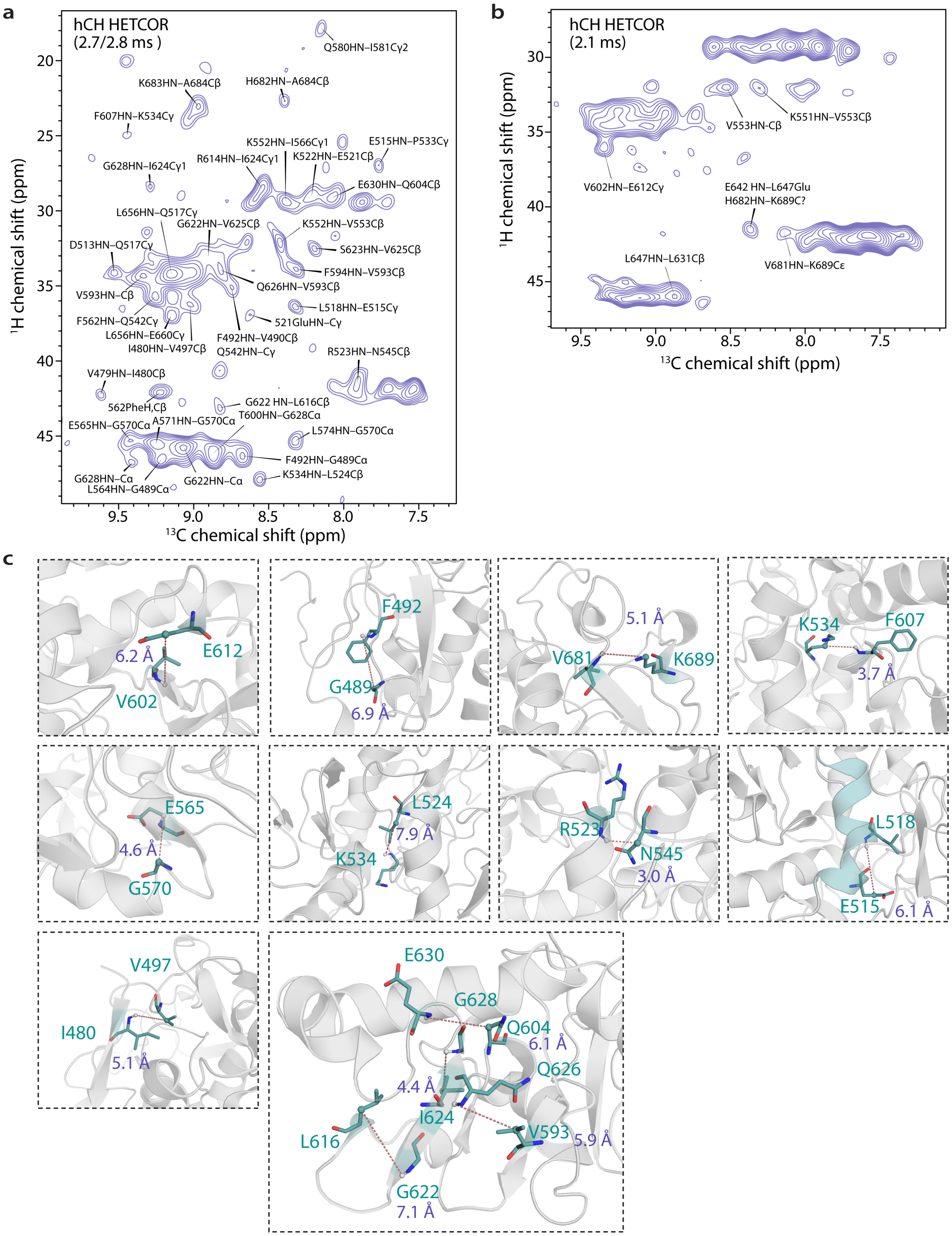

Supplementary Figure 11 | Intramolecular long-range C-H contacts in TPX2$^{\alpha5\text{-}\alpha7}$ protein on microtubules. **a**, **b** 2D CH HETCOR MAS NMR spectra of deuterated TPX2$^{\alpha5\text{-}\alpha7}$/MT assemblies. Intra and inter-residue correlations between amide ^{1}H and backbone or sidechain ^{13}C resonances were identified. The spectra were acquired at 20.0 T and a MAS rate of 60 kHz. The CP contact times were 2.7-2.8 ms for **a** and 2.1 ms for **b**. **c** Mapping of inter-residue ^{13}C-^{1}H distances on the MAS NMR structure of TPX2$^{\alpha5\text{-}\alpha7}$ bound to MTs. These medium and long-range restraints in TPX2$^{\alpha5\text{-}\alpha7}$ typically correspond to interatomic distances of 3-8 Å in the TPX2$^{\alpha5\text{-}\alpha7}$ structure. The identified correlating residues and atoms are shown in sticks and spheres, respectively.

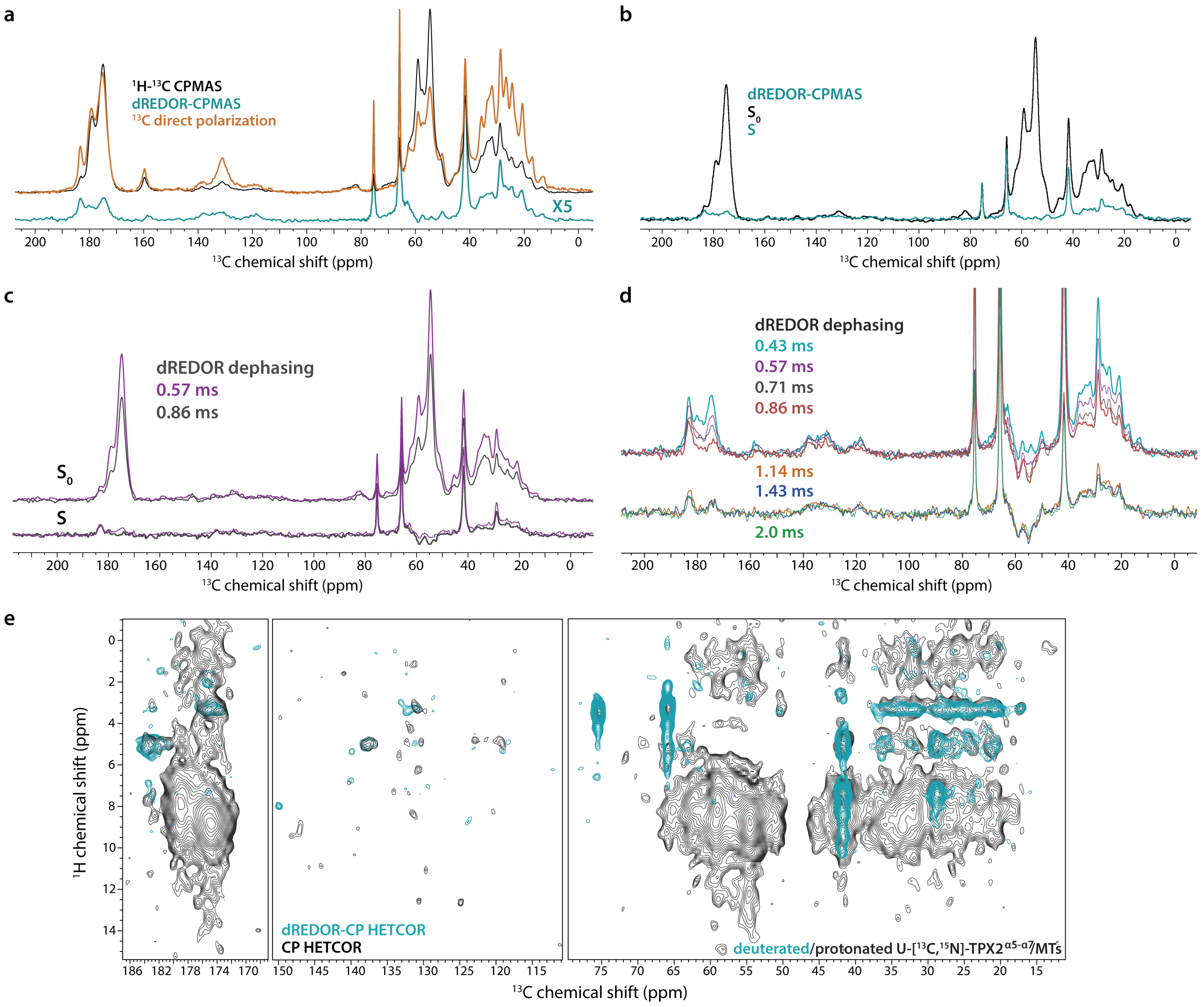

Supplementary Figure 12 | Dipolar-filtered MAS NMR spectra of deuterated TPX2$^{\alpha5\text{-}\alpha7}$/MT assemblies. **a** 1D ^{13}C direct polarization (orange), ^{1}H-^{13}C (black) and dREDOR-filtered (cyan) CPMAS spectra. The strong signals at ^{13}C chemical shifts of 66 and 76 ppm arise from solvent. **b** 1D ^{1}H-^{13}C dREDOR-CPMAS spectrum (cyan, S) and the control spectrum with zero-quantum relaxation (black, S_0). The spectra were acquired with a dephasing time of 0.43 ms and the same relaxation time, respectively. Intense Cα signals (44-64 ppm), which are present in the control spectrum, are completely dephased with dREDOR dipolar filters. **c**, **d** 1D dREDOR-CPMAS spectra with dephasing times ranging from 0.43 ms (6 rotor cycles) to 2.0 ms. The signals of directly ^{13}C and ^{15}N-bonded protons were suppressed with dREDOR dephasings of 0.43 ms or longer. The S_0 control spectra shown in **c** were acquired with the same relaxation duration as dREDOR dephasing times of 0.86 ms (black) and 0.57 ms (purple) for S spectra. **e** Overlay of 2D ^{1}H-^{13}C HETCOR spectrum(gray) with dREDOR-CP-based HETCOR spectrum (0.43 ms dephasing, cyan).

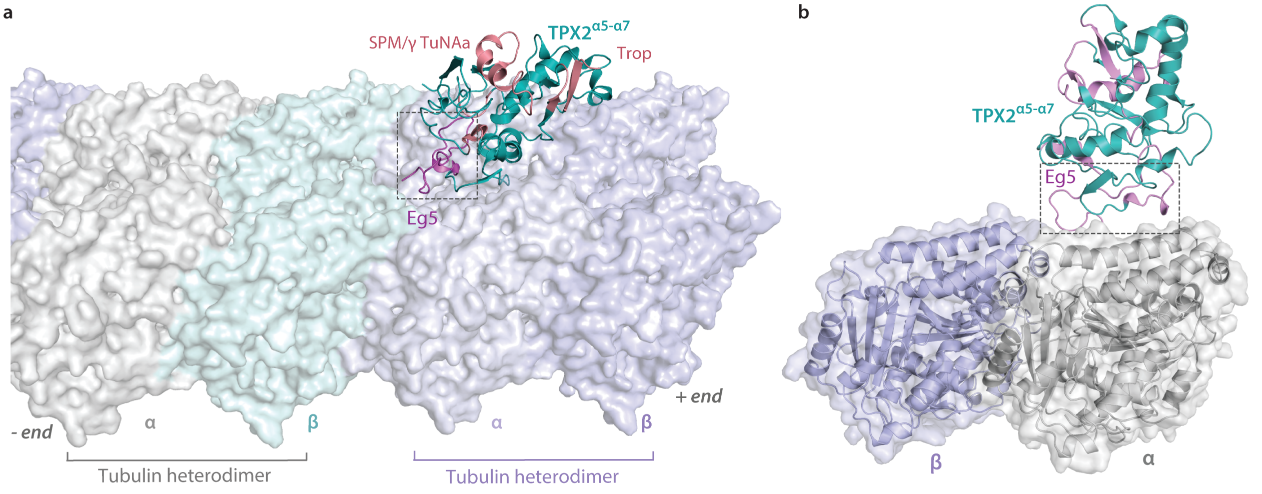

Supplementary Figure 13 | The minor binding mode for TPX2$^{\alpha5\text{-}\alpha7}$ interacting with MTs from the molecular modeling. This binding mode was predicted in **a** blind docking on polymerized microtubules and **b** restraints-guided docking on βα tubulin interdimer, with substantial less populations than the predominant binding mode. The minor binding mode is ruled out since it contradicts prior experimental evidence showing that the removal of Eg5 domain does not significantly reduce the binding affinity of TPX2$^{\alpha5\text{-}\alpha7}$ with MTs. The Eg5 binding domain (magenta) is indicated in dashed squares. Other functional regions and motifs of interests are colored in salmon in **a** and light magenta in **b**.

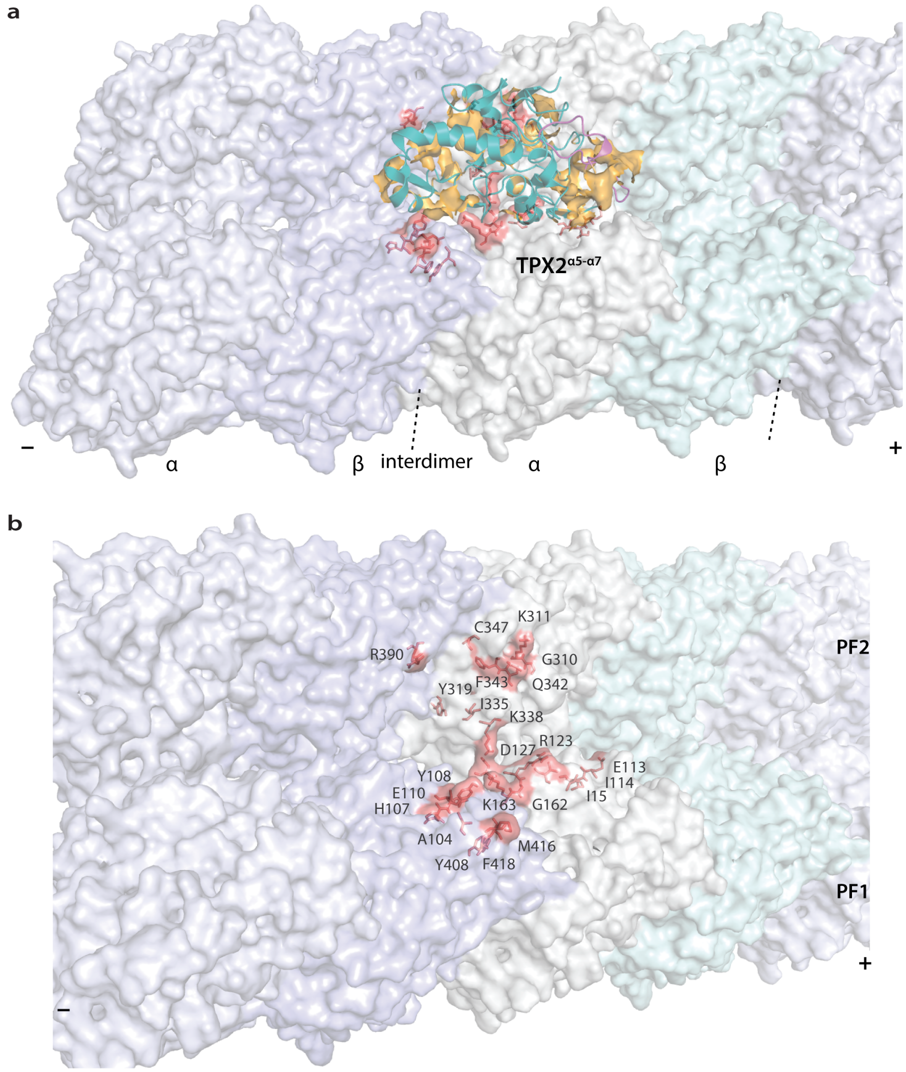

Supplementary Figure 14 | Intermolecular interface for TPX2$^{\alpha5\text{-}\alpha7}$-MTs interaction. a Residues of TPX2$^{\alpha5\text{-}\alpha7}$ and MTs at the binding interfaces (colored in orange and salmon, respectively), derived from dREDOR-based MAS NMR experiments. **b** Interface residues (salmon) of MTs that likely interact with TPX2$^{\alpha5\text{-}\alpha7}$. These interaction hotspots were identified using tentative assignments for ^{1}H resonances of tubulin based on SHIFTX2 prediction.

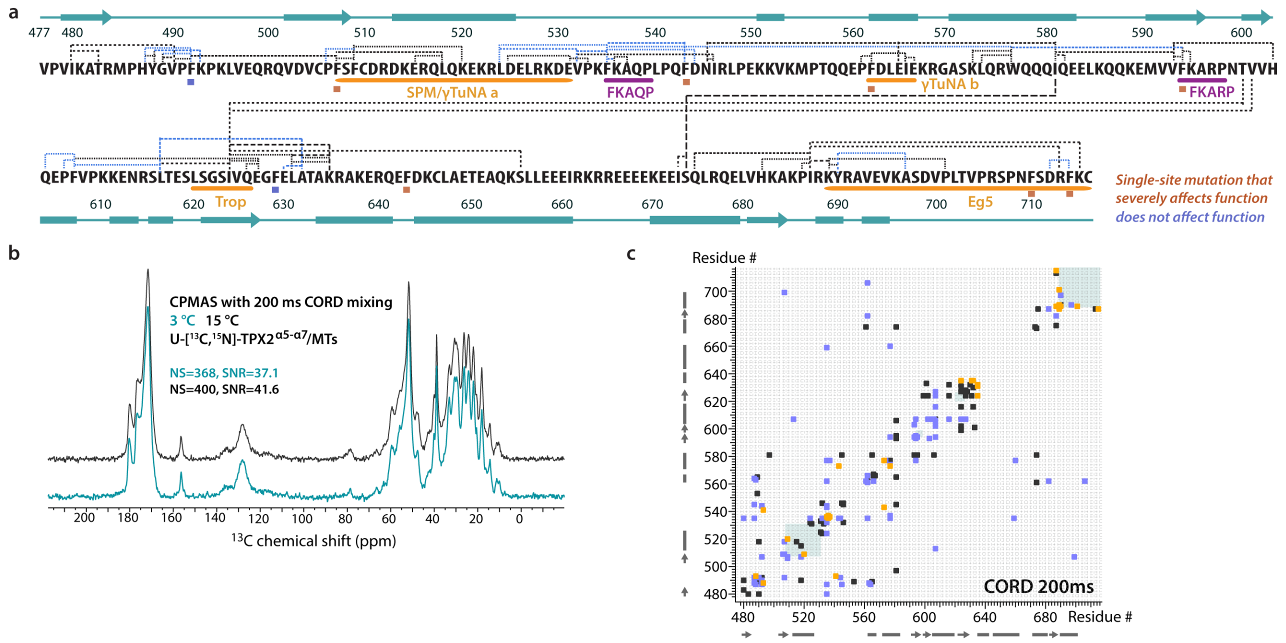

Supplementary Figure 15 | Dynamic residues and intramolecular contacts in TPX2$^{\alpha5\text{-}\alpha7}$ bound to MTs affected by temperature changes. a Mapping of inter-residue contacts (dashed lines) onto TPX2$^{\alpha5\text{-}\alpha7}$ sequence. These correlations are absent in the MAS NMR spectra acquired at the sample temperature of 15 °C. The Phe residues where single-site mutations severely affect TPX2 activity in stimulating microtubule nucleation and those having no effect function are indicated by brown and cyan dots, respectively. **b** Comparison of the 1D traces of ^{1}H-^{13}C CP-based CORD spectra acquired at 3 °C (cyan) and 15 °C (black). Both spectra were acquired with 200 ms homonuclear CORD mixing. The signal-to-noise ratios were 37.1 and 41.6 for the spectra acquired at 3 °C and 15 °C, respectively. There is no substantial difference in the spectral resolution and sensitivity of the two spectra. **c** Contact map of the intra- and inter-residue restraints in TPX2$^{\alpha5\text{-}\alpha7}$ bound to MTs that disappear in the CORD spectra at 15 °C. Contacts involving aromatic residues are color coded in purple; those involving dynamic lysine sidechains are shown in orange; others are shown in black. Functional regions are marked by rectangles in light cyan. Secondary structures of TPX2$^{\alpha5\text{-}\alpha7}$ derived from secondary chemical shifts are indicated on the side; rectangular bars and arrows represent helices and sheets, respectively.

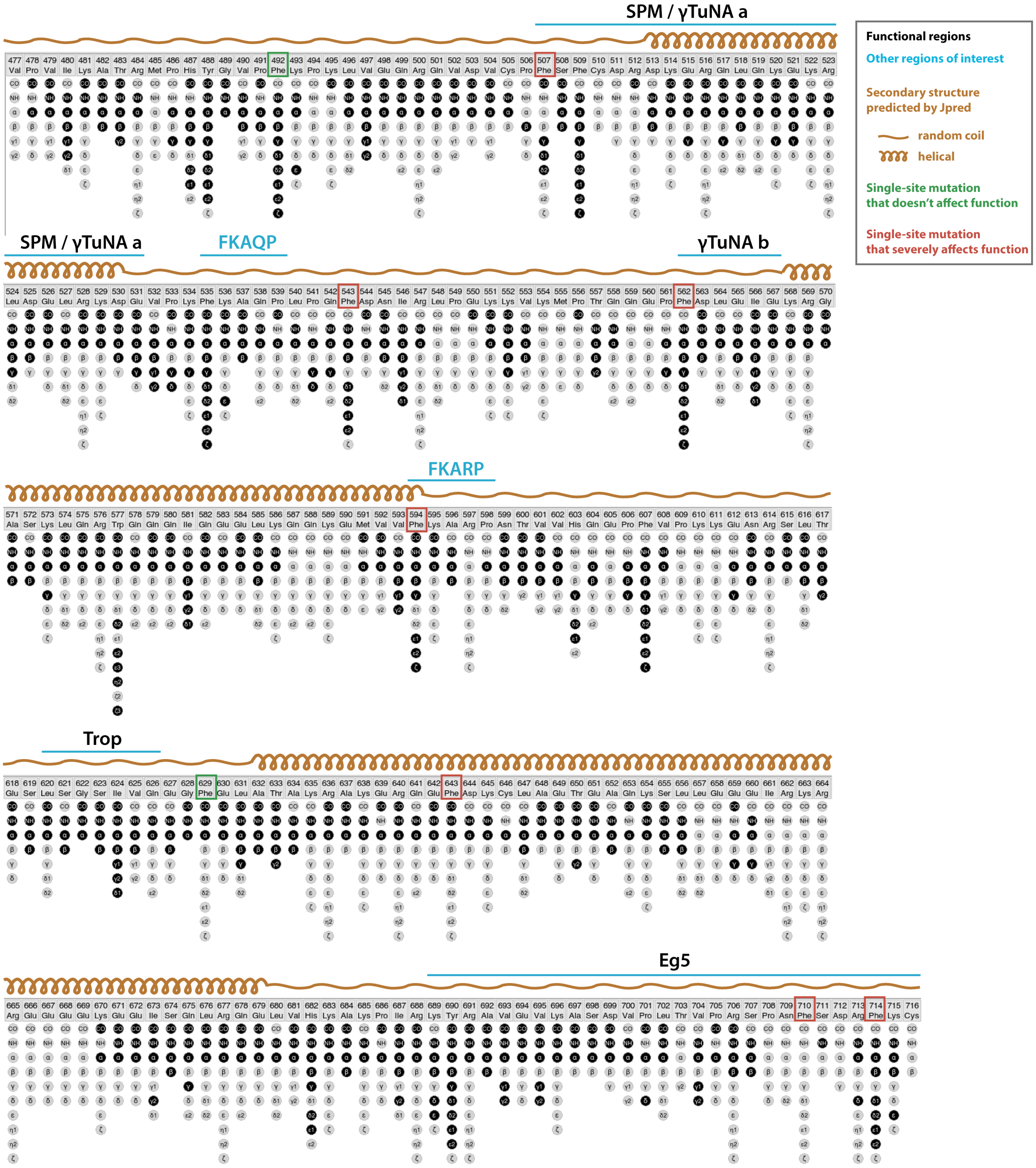

Supplementary Figure 16 | Summary of chemical shift assignments of TPX2$^{\alpha5\text{-}\alpha7}$ assembled with MTs. Assigned backbone and sidechain atoms are marked in black. The functional regions and secondary structures predicted by sequence-based Jpred are indicated on the top.

Supplementary Tables

Supplementary Table 1 | ^{1}H and ^{15}N line widths of representative peaks in the 2D ^{1}H-^{15}N HSQC solution NMR spectra of TPX2$^{\alpha5-\alpha7}$ in monodispersed solution and condensed form

	**Residue**	**δ^{1}H (ppm)**	**δ^{15}N (ppm)**	**Line widths (Hz) ^{1}H/^{15}N**		**Level of line broadening**
				Monodispersed	Liquid droplets	^{1}H Line widths ratio of LLPS: monodispersed
**1**	**S623**	8.3	116.4	16 / 13	18.9 / 20.9	1.2
**2**	**G570**	8.2	108.9	18.7 / 14.3	29.5 / 18.4	1.6
**3**	**G628**	8.4	109.6	16.1/ 13.2	27.6 / 32.7	1.7
**4**	**G622**	8.2	110.3	18 / 13.3	34.7 / 19.8	1.9
**5**	**T633**	7.9	114	19.3 /14.3	32.6 / 27.3	1.7
**6**	**T650**	8.0	114	18 / 14.2	25.8 / 24	1.4
**7**	**D644**	8.4	120	19 / 15.2		
**8**	**L702**	7.8	121.9	13.7 /15.7		
**9**	**A632/634**	8.0	121.9	15.9 /14.2	57.8 / 30.9	3.6
**10**	**F714**	7.9	122.4	16.6 / 15.8		
**11**	**L647**	8.3	122.3	13.4 / 15	15.6 / 38.4	1.2
**12**	**F607**	7.8	125.6	16.6 / 11.8	18.6 / 22.6	1.1
**13**	**A692/V479 #1**	8.3	124.3	15.8 / 15.3	26.8 / 33.1	1.7
**14**	**A692/V479 #2**	7.8	124.2	14.1 / 12.1		
**15**	**A692/V479 #3**	7.4	124.2	9.6 / 10.1	25.7 / 25.3	2.7
**16**	**K586/N599**	8.2	124.5	15.5 / 14.1	22.4 / 30.3	1.4
**17**	**unassigned #1**	8.0	124.2		23.8 / 23.8	
**18**	**unassigned #3**	8.2	119.4	14.6 / 13.5	23.5 / 28.5	1.6
**19**	**unassigned #4**	7.9	121.2	17.9 / 15.6	32 / 49.8	1.8
**20**	**V601/N545/E583 /A596 #1**	8.3	125.7	15.1 / 17.2	18.6 / 20.4	1.2
**21**	**V601/N545/E583 /A596 #2**	8.2	125.3	18.1 / 14.7	43.8 / 23.5	2.4
**22**	**V601/N545/E583 /A596 #3**	8.1	125.3	19.8 / 12.8	20.1 / 21.2	1.0

Supplementary Table 2 | Sensitivity of the ^{1}H-detected MAS NMR experiments on deuterated TPX2$^{\alpha5\text{-}\alpha7}$/MT using the 0.7 mm HCND and 1.3 mm HCN MAS probes.

MAS NMR rotor	Sample weights	2D hCH Experiments*				
		MAS frequency (kHz)	Transients	Absolute Sensitivity	Relative sensitivity	Relative sensitivity per unit mass of sample
0.7 mm	ca. 0.8 mg	110	192	19.7	1	2.8
1.3 mm	3.7 mg	60	160	31.7	1.61	1

*The spectra were acquired on the assemblies of deuterated (100% $^{1}H_N$ back-exchanged) TPX2$^{\alpha5\text{-}\alpha7}$ bound to natural abundant microtubules that were packed in 0.7 mm and 1.3 mm fast MAS Bruker rotors.

Supplementary Table 3 | Summary of MAS NMR experiments on TPX2$^{\alpha5\text{-}\alpha7}$/MT assemblies.

Experiments	Number of scans	S/N ratio (1st FID)	^{13}C-^{13}C mixing	Sample temperature	MAS Frequency	Experimental time
3.7 mg deuterated U-[^{13}C,^{15}N]-TPX2$^{\alpha5\text{-}\alpha7}$/n.a. MT, 1.3 mm rotor, 19.97 T						
hCH (long CP)	192	25.0		12 °C		18.5 h
hCH	160	31.7		12 °C		14.5 h
hNH	96	66.0		10 °C		10.5 h
hCANH	64	14.6		10 °C	60 kHz	3 d 11 h
hCONH	64	12.6		10 °C		4 d 9 h
h(CO)CA(CO)NH	512	6.5		10 °C		2 d 16.5 h
spRFDR#	608	5.6/16.3*		10 °C		3 d 19.5 h
1.0 mg deuterated U-[^{13}C,^{15}N]-TPX2$^{\alpha5\text{-}\alpha7}$/n.a. MT, 0.7 mm rotor, 19.97 T						
hCH	144	16.2		11 °C	100 kHz	1 d 15.5 h
hCH	192	19.7		15 °C	110 kHz	18 h
3.8 mg fully protonated U-[^{13}C,^{15}N]-TPX2$^{\alpha5\text{-}\alpha7}$/n.a. MT, 1.3 mm rotor, 14.1 T						
CORD	272	40.0 (CA)	50 ms	3 °C		1 d 19 h
	368	37.1	200 ms	3 °C		2 d 18 h
	400	41.6 (CA)	200 ms	15 °C		2 d 22 h
N(CA)CX	4096	19.7 (CA)	50 ms	15 °C	14 kHz	3 d 14 h
N(CO)CX	2048	26.2 (CA)	50 ms	15 °C		1 d 19 h
NCA	704	23.2		3 °C		17 h
HN HETCOR	512	18.9		3 °C		19.5 h
hCH	64	86.1		15 °C		11 h
hNH	128	117		15 °C	60 kHz	19 h
hCANH	128	16.8		15 °C		3 d 19 h
hCONH	128	12.8		15 °C		3 d 22.5 h

RFDR experiment with ^{13}C direct polarization

* The averaged signal-noise ratio is 5.6 for Cα resonances and 16.3 for aliphatic sidechain ^{13}C signals.

Supplementary Table 4 | Experimental parameters for ^{1}H-detected MAS NMR experiments on deuterated TPX2$^{\alpha5\text{-}\alpha7}$/MT assemblies.

Experiments	CP contact time			Radio frequency field strengths (kHz)						Acquisition time	
	1st CP	DCP	^{15}N-^{1}H or ^{13}C-^{1}H CP	1st CP		^{13}C-^{15}N DCP		^{13}C-^{1}H/^{15}N-^{1}H CP			
				^{1}H (linear)	^{13}C/^{15}N	^{13}C	^{15}N (tangent)	^{13}C/^{15}N	^{1}H (linear)	^{13}C	^{15}N
hCH (110 kHz MAS)	2.1 ms		2.1 ms	162	74			74	162	1.87 ms	
hCH (100 kHz MAS)	2.1 ms		2.1 ms	147	68			68	147		
hCH	2.1 ms		2.1 ms	40.7	116			116	40.7	2.1 ms	
hCH (long CP)	2.8 ms		2.7 ms	40.7	116			116	40.7	2.1 ms	
hNH	0.8 ms		0.6 ms	104	45			45	104		15 ms
hCANH	3.7 ms	14 ms	1.0 ms	103	47	50	35	45	101	3.6 ms	4.3 ms
hCONH	3.0 ms	10 ms	1.0 ms	56	10	45	37	45	101	3.7 ms	4.4 ms
h(CO)CA(CO)NH (60 kHz MAS)	3.8 ms	10 ms	1.9 ms	105	43	45	37	45	101	2.5 ms	2.4 ms

Supplementary Table 5 | Summary of sequential, medium- and long-range inter-residue distance restraints of TPX2$^{\alpha5\text{-}\alpha7}$ assembled on MTs derived from MAS NMR

<table>
<tr><th colspan="4">Long-range (|i – j| ≥ 5)</th></tr>
<tr><td>I480CB−L540CA</td><td>F535CE$^{\#}$−I480CG1</td><td>F594CG−W577CH2</td><td>E627CB−G622CA</td></tr>
<tr><td>A482CA–Q517CG</td><td>F535CE$^{\#}$−W577CE3</td><td>F594CG−W577CD2</td><td>G628CA−V602CB</td></tr>
<tr><td>H487CE1–N545CA</td><td>A537CB−E531CG</td><td>N599CA−N613CB</td><td>E630CA−L616CB</td></tr>
<tr><td>H487CE1–L564CA</td><td>P541CD−E521CB</td><td>V601CA−A596CA</td><td>L631CA−I624CD1</td></tr>
<tr><td>Y488CD$^{\#}$–K493CE</td><td>F543CD$^{\#}$−F535CZ</td><td>H603CG−V593CB</td><td>A632CB – V593CB</td></tr>
<tr><td>Y488CG–D563CA</td><td>D544CA−T557CG2</td><td>P606CA−N599CA</td><td>A632CA−L616CB</td></tr>
<tr><td>V490CB−L518CB</td><td>D544CA−F535CA</td><td>P606CA−I581CG2</td><td>S655CB−I624CG1</td></tr>
<tr><td>V490CB−I480CG2</td><td>N545CA−I581CB</td><td>F607CA−D513CB</td><td>S674CB−I581CB</td></tr>
<tr><td>F492CE$^{\#}$–H487CB</td><td>N545CA−E521CB</td><td>F607CB – K534CG</td><td>Q675CA−T703CG2</td></tr>
<tr><td>F492CE$^{\#}$−H487CD2</td><td>I546CG2−V532CA</td><td>F607CA−E612CG</td><td>Q675CG−I687CG2</td></tr>
<tr><td>F492CE$^{\#}$−F543CB</td><td>V553CB−G489CA</td><td>F607CZ−V602CB</td><td>H682CG−I687CB</td></tr>
<tr><td>F492CD$^{\#}$–D544CA</td><td>P561CG–S674CB</td><td>F607CE$^{\#}$−V602CB</td><td>H682CG−K689CD</td></tr>
<tr><td>K493CE–A537CB</td><td>F562CE$^{\#}$–R706CB</td><td>F607CE$^{\#}$−V601CB</td><td>H682CG−W577CZ3</td></tr>
<tr><td>K493CE−P541CA</td><td>E565CA–G489CA</td><td>F607CZ–E627CB</td><td>I687CG2−R713CD</td></tr>
<tr><td>V497CB−I480CG2</td><td>E565CB−I581CD1</td><td>F607CB – E649CA</td><td>I687CG2−H682CB</td></tr>
<tr><td>V497CB−A482CA</td><td>K573CA−F562CB</td><td>L616CB−I624CD1</td><td>K689CB−S674CB</td></tr>
<tr><td>F507CG–L518CB</td><td>K573CG−Q582CA</td><td>G622CA−E612CG</td><td>K689CB−A684CA</td></tr>
<tr><td>F507CD$^{\#}$–D699CA</td><td>W577CE2−F594CE$^{\#}$</td><td>I624CD1−K635CA</td><td>K689CE−A632CB</td></tr>
<tr><td>F509CB−P533CG</td><td>W577CD2–E660CG</td><td>I624CD1−N599CA</td><td>P701CA−K689CE</td></tr>
<tr><td>D513CB−P506CB</td><td>I581CD1−V497CG1</td><td>I624CD1−V602CB</td><td>F714CB−R706CB</td></tr>
<tr><td>D525CA−E531CG</td><td>V593CB−G622CA</td><td>I624CG2−V601CB</td><td>K715CE−I687CG2</td></tr>
<tr><td>V532CG1−F562CB</td><td>V593CB−D530CB</td><td>I624CD1−E531CB</td><td>F507CG−F607CG</td></tr>
<tr><td>F535CA−L540CA</td><td>F594CE$^{\#}$−F607CB</td><td>I624CD1−F607CB</td><td>I546CD1−K552CG</td></tr>
<tr><td>F535CE$^{\#}$−E659CG</td><td>F594CE$^{\#}$−I624CG1</td><td>I624CG2−F607CB</td><td>Y690CD$^{\#}$−W577CZ3</td></tr>
<tr><td>F535CG−H487CB</td><td>F594CE$^{\#}$ – E627CB</td><td>V625CB – L631CB</td><td></td></tr>
<tr><th colspan="4">Medium-range (1 < |i – j| < 5)</th></tr>
<tr><td>F492CE$^{\#}$−V490CB</td><td>P539CA−A537CB</td><td>W577CD2−L574CA</td><td>E627CB−I624CG2</td></tr>
<tr><td>F509CG−P506CB</td><td>F543CE$^{\#}$−N545CB</td><td>V601CA−N599CA</td><td>L631CA−T633CA</td></tr>
<tr><td>F509CZ−F507CD$^{\#}$</td><td>F543CE$^{\#}$−P541CG</td><td>H603CE1−F607CE$^{\#}$</td><td>A632CA−E630CA</td></tr>
<tr><td>E515CG−L518CB</td><td>F562CD$^{\#}$−E565CA</td><td>F607CG–Q604CA</td><td>A632CB–K635CA</td></tr>
<tr><td>E531CB−P533CD</td><td>F562CD$^{\#}$–E565CB</td><td>F607CD$^{\#}$–Q604CB</td><td>Y488CG−V490CB</td></tr>
<tr><td>E531CG−P533CD</td><td>F562CE$^{\#}$–E565CB</td><td>S621CB−I624CG1</td><td>F507CD$^{\#}$−F509CB</td></tr>
<tr><td>F535CE$^{\#}$−E531CA</td><td>F562CE$^{\#}$−L564CA</td><td>I624CD1−E627CA</td><td>F509CG−F507CG</td></tr>
<tr><td>F535CE$^{\#}$–V532CB</td><td>F562CG−I566CB</td><td>I624CD1−E627CB</td><td>Y690CG−R688CA</td></tr>
<tr><th colspan="4">Short-range (|i – j| = 1)</th></tr>
<tr><td>H487CE1–Y488 CA</td><td>F543CB−Q542CG</td><td>F594 CG–K595 CA</td><td>H487CG−P486CG</td></tr>
<tr><td>H487CG−Y488CE$^{\#}$</td><td>D544CA−F543CB</td><td>F607CZ−P606CB</td><td>F492CZ−K493CA</td></tr>
<tr><td>V490CB−G489CA</td><td>N545CA−I546CA</td><td>G628CA−E627CB</td><td>F509CG−S508CA</td></tr>
<tr><td>F509CE$^{\#}$−S508CA</td><td>F562CE$^{\#}$−P561CG</td><td>A632CA−T633CA</td><td>F535CE$^{\#}$−K534CG</td></tr>
</table>

F535CE#−K534CA	F562CD#−P561CG	S674CB−I673CG2	E565CB−I566CD1
F535 CE#−K536 CE	I566CB−E567CA	H682CG−K683CA	H603CG−Q604CA
P541CA−L540CA	W577CD2−K573CA	Y690CD#−K689CA	Y690CG−K689CE
Q542CG−P541CD	F594CE#−V593CA	F714CD#−R713CA	

Supplementary Table 6 | MAS NMR distance and dihedral constraints

Distance constraints ^{13}C-^{13}C	
Unambiguous	340
Intra-residue	172
Inter-residue	169
Sequential (|*i*-*j*| = 1)	32
Medium-range (1 ≤ |*i*-*j*| < 5)	35
Long-range (|*i*-*j*| ≥ 5) (sidechain-sidechain)	101 (62)
Ambiguous	1
**Total ^{13}C-^{13}C restraints**	**341**
Summary of dihedral angle restraints	
ϕ	144
ψ	144
Structure statistics from 10 lowest energy subunits	
Violations (mean ± s.d.)	
Distance restraints ≥ 7.2 Å (Å)	0.959 ± 0.011
Dihedral angle restraints ≥ 5° (°)	27.872 ± 0.189
Max. distance restraint violation (Å)	5.678
Max. dihedral angle restraint violation (°)	106.663
Deviations from idealized geometry	
Bond lengths (Å)	0.021 ± 0.000
Bond angles (°)	2.546 ± 0.014
Impropers (°)	57.678 ± 0.004
Average pairwise r.m.s.d. (Å)	
Heavy	3.31 ± 0.48
Backbone (N, Cα, C)	2.54 ± 0.45

Supplementary Table 7 | TPX2$^{\alpha5\text{-}\alpha7}$ residues detected by dREDOR-based MAS NMR experiments

**In close contact with MTs**		**Others**
A482	P606	I480
H487	F607	C505
Y488	E612	F507
P491	N613	E515
D513	T617	A537
K520	V625	T650
E521	E627	E651
L524	L631	E660
I546	R636	
P541	H682	
K552	A684	
T557	K689	
F562	Y690	
I566	A692	
L574	V695	
I581	R706	
V601	S707	
V602	F714	
H603	K715	

Supplementary Table 8 | Tentative assignments for the ^{1}H resonances of microtubules in the 2D dREDOR-filtered HETCOR MAS NMR spectrum

δ^{1}H (ppm)	Resonance	Chain*	Protofilament#
0.6	I115 HG	A	PF1
1.4	K338/K163 HG	A	PF1
1.7	A104 HB2	B	PF1
2.0	I114/V159 HB	A	PF1
2.3	H107 HB2	B	PF1
2.4	E113 HG	A	PF1
2.5	M416 HG	B	PF1
2.8	D127 HB	A	PF1
3.4	I335 HA	A	PF1
3.5	T191 HA	A	PF1
6.9	Y108/408/418 HD	B	PF1
2.2	Q342 HG	A	PF2
4.6	R390 HA	B	PF2
5.1	K311 HA	A	PF2
4.9	F418 HA	B	PF1
	S439/C347 HA	A	PF2
5.6	Y319 HA	A	PF2
7.4	Q342 HE2	A	PF2
8.3	E110 HN	B	PF1
	G310 HN	A	PF2
	R123 HN	A	PF1

*: A and B chain denote to α and β tubulin, respectively.
#: PF1 and PF2 denote to neighboring protofilaments of MTs (indicated in Figure S12).
