# Supplementary material for: Structural basis of protein condensation on microtubules underlying branching microtubule nucleation
Source: Nat Commun. 2023 Jun 21;14:3682. doi: 10.1038/s41467-023-39176-z (PMC10284871; doi:10.1038/s41467-023-39176-z)
Supplement: Supplementary file 2 — Reporting Summary [file 41467_2023_39176_MOESM2_ESM.pdf]

nature portfolio

Corresponding author(s): Tatyana Polenova, Sabine Petry

Last updated by author(s): May 22, 2023

Reporting Summary

Nature Portfolio wishes to improve the reproducibility of the work that we publish. This form provides structure for consistency and transparency in reporting. For further information on Nature Portfolio policies, see our Editorial Policies and the Editorial Policy Checklist.

Statistics

For all statistical analyses, confirm that the following items are present in the figure legend, table legend, main text, or Methods section.

n/a	Confirmed	
☐	☒	The exact sample size (*n*) for each experimental group/condition, given as a discrete number and unit of measurement
☒	☐	A statement on whether measurements were taken from distinct samples or whether the same sample was measured repeatedly
☒	☐	The statistical test(s) used AND whether they are one- or two-sided<br>*Only common tests should be described solely by name; describe more complex techniques in the Methods section.*
☒	☐	A description of all covariates tested
☒	☐	A description of any assumptions or corrections, such as tests of normality and adjustment for multiple comparisons
☒	☐	A full description of the statistical parameters including central tendency (e.g. means) or other basic estimates (e.g. regression coefficient) AND variation (e.g. standard deviation) or associated estimates of uncertainty (e.g. confidence intervals)
☒	☐	For null hypothesis testing, the test statistic (e.g. *F*, *t*, *r*) with confidence intervals, effect sizes, degrees of freedom and *P* value noted<br>*Give P values as exact values whenever suitable.*
☒	☐	For Bayesian analysis, information on the choice of priors and Markov chain Monte Carlo settings
☒	☐	For hierarchical and complex designs, identification of the appropriate level for tests and full reporting of outcomes
☒	☐	Estimates of effect sizes (e.g. Cohen's *d*, Pearson's *r*), indicating how they were calculated

Our web collection on statistics for biologists contains articles on many of the points above.

Software and code

Policy information about availability of computer code

Data collection	NMR data were collected using Bruker TopSpin version 3.5 and 4.0, a standard commercial program.
Data analysis	NMR data were processed with Bruker TopSpin (version 4.1.3) and NMRPipe (version mac). NMR spectra were visualized and analyzed using NMRFAM-SPARKY (version 3.115), CcpNmr Analysis (version 2.4), and MestReNova (14.1.1). Backbone dihedral angel restraints were generated from experimental NMR data using TALOS-N. MAS NMR structure of TPX2 was calculated and refined using X-PLOR NIH (version 2.53).<br>Models based on sequence-based predictions were generated using Robetta and AlphaFold2.<br>Structural models for TPX2 binding with microtubules were generated from molecular dockings using ClusPro (version 2.036) and HADDOCK (version 2.4).<br><br>Coordinate transformation to generate the structure of the TPX2 were accomplished with in-house UCSF Chimera python scripts (UCSF Chimera version 1.13).<br>Restraint tallying and format conversions were carried out with in-house Python 2.7 scripts.<br>Structure ensembles were rendered for visualization in PyMOL 1.8.4.1 using in-house shell/bash scripts for batch rendering.<br>Secondary structure elements were classified according to TALOS-N and manual inspection.<br>RMSD values were calculated using routines in the Xplor-NIH (version 2.53).<br>Multi-color images were acquired using NIS-Elements software (Nikon).

For manuscripts utilizing custom algorithms or software that are central to the research but not yet described in published literature, software must be made available to editors and reviewers. We strongly encourage code deposition in a community repository (e.g. GitHub). See the Nature Portfolio guidelines for submitting code & software for further information.

Data

Policy information about availability of data

All manuscripts must include a data availability statement. This statement should provide the following information, where applicable:

- Accession codes, unique identifiers, or web links for publicly available datasets
- A description of any restrictions on data availability
- For clinical datasets or third party data, please ensure that the statement adheres to our policy

The MAS NMR structure of TPX2 bound to microtubules and the coordinates have been deposited at the Protein Data Bank with accession code PDB ID 8CX6. The MAS NMR chemical shifts are deposited at the Biological Magnetic Resonance Bank (BMRB) under BMRB entry ID 31025. The cryo-EM structure of paclitaxel-stabilized microtubules used in this study is available under PDB 3J6G.

Human research participants

Policy information about studies involving human research participants and Sex and Gender in Research.

Reporting on sex and gender	N/A
Population characteristics	N/A
Recruitment	N/A
Ethics oversight	N/A

Note that full information on the approval of the study protocol must also be provided in the manuscript.

Field-specific reporting

Please select the one below that is the best fit for your research. If you are not sure, read the appropriate sections before making your selection.

☒ Life sciences ☐ Behavioural & social sciences ☐ Ecological, evolutionary & environmental sciences

For a reference copy of the document with all sections, see nature.com/documents/nr-reporting-summary-flat.pdf

Life sciences study design

All studies must disclose on these points even when the disclosure is negative.

Sample size	The sample size were chosen based on the size and sample volume of magic-angle spinning rotors for different types of MAS NMR experiments. Multiple samples of isotopically labeled recombinant protein were prepared and packed into 1.3 mm and 0.7 mm MAS rotors, which have capacities of 3 ul and 0.6 ul, respectively.
Data exclusions	No data were excluded from the analyses.
Replication	The measurements were replicated at least three times with similar results.
Randomization	This study does not include any clinical trial or research.
Blinding	This study does not include any clinical trial or research.

Reporting for specific materials, systems and methods

We require information from authors about some types of materials, experimental systems and methods used in many studies. Here, indicate whether each material, system or method listed is relevant to your study. If you are not sure if a list item applies to your research, read the appropriate section before selecting a response.

Materials & experimental systems

n/a	Involved in the study
☐	☒ Antibodies
☒	☐ Eukaryotic cell lines
☒	☐ Palaeontology and archaeology
☒	☐ Animals and other organisms
☒	☐ Clinical data
☒	☐ Dual use research of concern

Methods

n/a	Involved in the study
☒	☐ ChIP-seq
☒	☐ Flow cytometry
☒	☐ MRI-based neuroimaging

Antibodies

Antibodies used

anti-biotin monoclonal antibody (Thermo Fisher, #03-3700)

Validation

The biotin monoclonal antibody is validated in chemical for applications in immunocytochemistry (ICC/IF).
Host/Isotope: Mouse/ IgG1. Clone: Z021. Immunogen: Synthetic biotin.
References: Chamma et.al Nat. Commun. 7:10773 (2016) DOI: 10.1038/ncomms10773
Boettiger et. al Cell Rep. 3(1):8-15 (2013) DOI: 10.1016/j.celrep.2012.12.015
